# Supplementary material for: Binge drinking and associated factors among school students: a cross-sectional study in Zhejiang Province, China
Source: BMJ Open. 2018 Apr 12;8(4):e021077. doi: 10.1136/bmjopen-2017-021077 (PMC5898305; doi:10.1136/bmjopen-2017-021077)
Supplement: Author's manuscript [file bmjopen-2017-021077.draft_revisions.pdf]

BMJ Open

BMJ Open is committed to open peer review. As part of this commitment we make the peer review history of every article we publish publicly available.

When an article is published we post the peer reviewers' comments and the authors' responses online. We also post the versions of the paper that were used during peer review. These are the versions that the peer review comments apply to.

The versions of the paper that follow are the versions that were submitted during the peer review process. They are not the versions of record or the final published versions. They should not be cited or distributed as the published version of this manuscript.

BMJ Open is an open access journal and the full, final, typeset and author-corrected version of record of the manuscript is available on our site with no access controls, subscription charges or pay-per-view fees (http://bmjopen.bmj.com).

If you have any questions on BMJ Open's open peer review process please email info.bmjopen@bmj.com

Binge Drinking and Associated Factors among Adolescent Students in China

Journal:	*BMJ Open*
Manuscript ID	bmjopen-2017-021077
Article Type:	Research
Date Submitted by the Author:	12-Dec-2017
Complete List of Authors:	Wang, Hao; Zhejiang Provincial Center for Diseases Control and Prevention, Department of NCDs Control and Prevention<br>Hu, Ru-Ying; Zhejiang Provincial Center for Diseases Control and Prevention, Department of NCDs Control and Prevention<br>Zhong, Jie-Ming; Zhejiang Provincial Center for Diseases Control and Prevention, Department of NCDs Control and Prevention<br>Du, Huaidong; University of Oxford, Clinical Trial Service Unit & Epidemiological Studies Unit (CTSU), Nuffield Department of Population Health; University of Oxford, Medical Research Council Population Health Research Unit, Nuffield Department of Population Health<br>Wang, Meng; Zhejiang Provincal Center for Disease Control and Prevention, Department of NCDs Control and Prevention<br>Yu, Min; Zhejiang Provincial Center for Diseases Control and Prevention
Keywords:	Binge drinking, Alcohol, Adolescents, Factors

SCHOLARONE™
Manuscripts

For peer review only

Binge Drinking and Associated Factors among Adolescent Students in China

Hao Wang[1], Ruying Hu[1], Jieming Zhong[1], Huaidong Du[2, 3], Wang Meng[1], Min Yu[1]

Author affilitations

1. Department of NCDs Control and Prevention, Zhejiang Provincial Center for Diseases Control and Prevention, Hangzhou, China

2. Clinical Trial Service Unit & Epidemiological Studies Unit (CTSU), Nuffield Department of Population Health, University of Oxford, Oxford, UK

3. Medical Research Council Population Health Research Unit, Nuffield Department of Population Health, University of Oxford, Oxford, UK

Correspondence to Min Yu; myu@cdc.zj.cn

For peer review only

For peer review only - http://bmjopen.bmj.com/site/about/guidelines.xhtml

For peer review only

Abstract

Objective: To investigate the prevalence and correlates of binge drinking among adolescents in Zhejiang, China.

Methods: A school-based survey was performed between April and May 2017. 23 543 adolescents from 442 different schools were surveyed using an anonymous self-administered questionnaires. Multivariate logistic regression models were used for data analyses.

Results: The mean (SD) age of the participants was 15.6 years (1.7) and 51.3% of them were boys. One out of five adolescents reported to drink in the past 30 days and one out of ten adolescents reported binge drinking during the past month. In comparison with middle school and academic high school, the binge drinking prevalence in vocational high school was the highest. Older age, bad academic performance, physical activity, screen time, loneliness, insomnia, suicide attempt, smoking, fighting, being bullied, sexual experience were found to be positively associated with adolescent binge drinking.

Conclusions: Binge drinking is common among adolescents in Zhejiang China. Comprehensive alcohol measures, including raising alcohol prices, reducing alcohol outlet density, and banning alcohol advertisement, are urgently needed.

Keywords: Binge drinking; Alcohol; Adolescents; Factors

For peer review only - http://bmjopen.bmj.com/site/about/guidelines.xhtml

Strengths and limitations of this study

- This was a school-based study with relatively large sample size, high response rate and representative provincial samples from China.
- The study questionnaire involved a great number of factors, which might help us better understand how to prevent adolescent binge drinking.
- The study was based on a cross-sectional study, which did not allow us establish the temporal relationship between factors and binge drinking.
- Only school-going adolescents participated in the survey. Students who was expelled, suspended, or dropped out were excluded from the survey. If those were more likely to binge drinking, this may lead to underestimate the overall prevalence.

For peer review only

Introduction

Alcohol consumption has been identified as an important risk factor for chronic disease and injury [1]. Alcohol consumption attributable DALYs (disability-adjusted life years) have increased by more than 25% over the years 1990–2016, accounting for an estimated 99.2 million DALYs worldwide. In 2016, alcohol consumption is responsible for more than 2.8 million deaths worldwide, including approximately 0.7 million in China[2].

Many studies have clearly demonstrated that alcohol use during early adolescence is a risk factor for the later development of alcohol dependence [3-7]. Some reported risk factors for binge drinking among adolescents include smoking, illicit drug use, sexual behaviours[3 8-10]. However, most of the research came from western countries. Little is known about the pattern of binge drinking and its associated factors in China. Prevalence of binge drinking among adolescents varied across different countries, with 8% in Iceland and 56% in Denmark[11]. Among 25 European countries, prevalence of binge drinking among adolescents rose from 36% in 1995 to 42% in 2007, and then declined to 35% in 2015[11]. In the United States, the corresponding figure declined from 31.3% in 1991 to 17.7% in 2015[12]. An up-to-date study, surveyed 13 811 high school students from China's 3 metropolises in 2013, found the prevalence of binge drinking was 4.8% [13]. Due to some reason, not all of the grades were covered, yielding an underestimation of prevalence. Therefore, the current study was designed with an aim to examine the prevalence of binge drinking and its correlate factors among adolescents in Zhejiang Province, China.

Methods

Survey design

The survey was a two-stage sampling design. In stage one, 30 counties, including 12 urban areas and 18 rural areas, were sampled from all 90 counties of Zhejiang Province. In stage two, 10 classes of middle school, 5 classes of academic high school, and 5 classes of vocational high school were selected randomly within each chosen counties, respectively. All the students in the chosen classes were eligible to participate. The survey questionnaire was modelled from existing surveys including Youth Risk Behaviour Survey conducted by the Centers for Disease Control and Prevention[14] and the international Global School-based Student Health Survey supported by the World Health Organization[15]. Survey questions addressed demographic characteristics (including age, gender, parental educational level, parental marital status, number of siblings etc.), tobacco and alcohol use, physical activity, violence, injuries, suicidal behaviours, sexual behaviours. The survey was conducted between April and May 2017. Subjects filled in the anonymous self-administrated questionnaire in the classrooms. The finished questionnaires were handed in on the spot.

For peer review only

For peer review only - http://bmjopen.bmj.com/site/about/guidelines.xhtml

Measures

Alcoholic drinks include beer, wine, wine coolers, and liquor such as rum, gin, vodka, or whiskey. Current drinking was accessed through the question: "During the past 30 days, on how many days did you have at least one drink of alcohol? (Options include: 0 days, 1-2 days, 3-5 days, 6-9 days, 10-19 days, 20-29 days, and 30 days)." Participants were considered as current drinkers if they answered had drunk at least 1 day during the past 30 days. Binge drinking was by assessed the question: "During the past 30 days, on how many days did you have 4 or more drinks of alcohol in a row (if you are female) or 5 or more drinks of alcohol in a row of 1 to 2 hours (if you are male)? (0 days, 1 days, 2 days, 3-5 days, 6-9 days, 10-19 days, ≥20 days)." Participants were defined as binge drinkers if they answered at least 1 day. Other variables were described in Table 1.

Quality Control

The survey was conducted by local Center for Disease Control and Prevention. All of the surveyors were trained before the survey. In order to improve response rate, every recruited student was given a pencil box as a gift. The survey was anonymous, and without filling in the participants' name on the questionnaire.

Ethics Statement

This study was approved by the ethics committee of Zhejiang Provincial Centre for Disease Control and Prevention. Written informed consent was given before survey, and obtained from all participants and their guardians. The ethics committee approved this procedure.

Statistical analysis

A weighting factor was applied to each student record to adjust for non-response and for the varying probabilities of selection. The weight used for estimation in this survey is given by: W= W1 * W2 * f1* f2. W1 = the inverse of the probability of selecting the county; W2= the inverse of the probability of selecting the classroom within the county. f1= a student-level nonresponse adjustment factor calculated by class. f2= a post-stratification adjustment factor calculated by grade[16].

Continuous variables were given as the mean ± standard deviation. The prevalence of current drinking and binge drinking was given as percent and 95% confidence intervals (CI). Categorical variables between groups were performed using Chi-square test. Weighted prevalence between groups was performed using Rao-Scott Chi-square test. Logistic regression was used to assess factors associated with binge drinking. All analyses were performed with SAS software V.9.3. All statistical tests were two tailed, and *P*-values <0.05 were considered statistically significant[17].

Results

Descriptive statistics

For peer review only - http://bmjopen.bmj.com/site/about/guidelines.xhtml

A total of 24157 students was in the chosen classes and due to missing or incompletion of questionnaires and refusal to participation, 614 students were excluded, yielding 23,543 eligible participants (response rate 97.5%) in the current analysis. Of which, 12 068 (51.3%) were boys and the average age was 15.6 years for both boys and girls. 12 207 (51.9%) of the participants were middle school students, 6 477 (27.5%) were academic high school students and 4 859 (20.6%) came from vocational high school .

Table 2 demonstrated that 43.6% boys and 59.9% girls had siblings, respectively. More girls ever had attempted suicide than boys (4.5% vs.2.8%). The percentages of boys who were in a physical fight and being bullied were 24.6% and 15.5%, respectively. While the corresponding figures were 6.5% and 10.2% for girls. Boys were more likely to have sexual experience than girls (4.9% vs.2.4%).

The prevalence of drinking

Just as shown in the Table 3, the prevalence of current drinking was 22.8%, higher in boys than in girls (27.0% vs. 18.2%). There was no statistical significance of current drinking prevalence between urban and rural (24.4% vs. 22.0 %). The prevalence of binge drinking was 9.3%, also higher in boys than in girls (11.8% vs. 6.5%). No significant difference was found for binge drinking prevalence between urban and rural areas (9.8% vs. 9.0%). The prevalence of binge drinking among middle school, academic high school, and vocational high school was 6.3%, 7.7% and 17.9%.

Logistic regression analysis

Table 4 showed that univariate and multivariate logistic regression analysis result of socio-demographic and health-related behaviors of binge drinking. Univariate analysis showed that age, types of school, parental marital status, paternal education level, screen time, loneliness, insomnia, suicide attempt, current smoking, fighting, being bullied, and sexual experience were significantly associated with binge drinking. Besides, physical activity was found to be significantly associated with binge drinking for boys.

After adjusted other variables in the model, multivariable analysis showed that comparing to middle school students, students in academic high school and vocational high school were more likely to binge drink. Students who spent more time on watching screen, felt insomnia, attempted suicide, being current smoker, being bullied and having sexual experience were more likely to binge drink. Girls who often or always felt lonely were 1.4 times more likely to binge drink than girls who never or occasionally felt lonely (OR=1.44, 95%CI： 1.05-1.96). Boys with bad academic performance in classes were 1.3 times more likely to binge drink than boys with excellent academic performance (OR=1.27, 95%CI：1.04-1.56)).

For peer review only - http://bmjopen.bmj.com/site/about/guidelines.xhtml

Discussion

In the present study, the overall prevalence of binge drink was 9.3%, which was lower than the majority of European countries, such as Denmark (56%), Netherlands (39%), Poland (35%), France (31%),Portugal (20%), but slightly higher than adolescents in Iceland (8%)[11] and Hong Kong (7.1%)[18]. We found the binge drinking prevalence was higher in boys than in girls, which was consistent with results from other studies[10 13 18], but different from those in several European countries (Denmark, Austria, etc.) where no sex difference observed[11]. The prevalence of binge drink among academic high school students was higher than that of middle school. The vocational high school had the highest prevalence of binge drinking, which means students of vocational high school should be target population of intervention. A possible explanation was that differ from middle school and academic high school students, vocational high school students are going to step into society after graduation, and so they maybe need more social communication. Meanwhile, drinking alcohol was considered as an important skill of social communication[19]. The relationship on parental educational level with binge drinking was not very clear in literature. No association was found between parental educational levels with binge drinking in our study. In a systematic review including 20 studies from 10 countries or areas, parental socioeconomic status (SES), defined as the educational level, income, or occupation, was weakly but positively associated with binge drinking in developing countries. However, such association was not found in developed countries[20]. Previous studies reported that non-intact family structure was associated with alcohol drinking due to low family attachment or insufficient parent-child communication[21 22]. Contrary to the expectations, no such association was found in our study. This may be related to somewhat different parental alcohol-specific rules. Van den et al. found that adolescents were less likely to drink under strict alcohol-specific rules at homes despite non-intact families[23].

In our study, nearly one out of five adolescents spent more than 4 hours each day screen time, i implying the more and more serious problem in China. Previous studies mainly focused on the relation between television watching with diseases or other factors[24-27]. However, with the exponential growth of electronic screen products, such measures of screen time may be underestimate screen time[28]. To our best knowledge, this is the first study to examine association between adolescent binge drinking and screen time. Our study showed that screen time was positively associated with binge drinking. In addition,substance use might be a means of self-medication and of alleviating the negative feelings that emanate from being lonely[29]. In our study, loneliness was positively associated with adolescent binge drinking, but this association was found only among girls, which accorded with findings among Arkhangelsk adolescents[30]. As demonstrated in our study, with the increase of insomnia, the odds of adolescent binge drinking increased. A previous study including 14 089 participants published by Popovici et al. revealed that binge drinking was positively associated with sleep problems (having trouble falling asleep or

For peer review only - http://bmjopen.bmj.com/site/about/guidelines.xhtml

staying asleep), independent of psychiatric conditions. There was a dose-response relationship between sleep problems and frequency of binge drinking[31], which was in accordance with our study. Rishi et al. found that binge drinking could reverse sleep-wake cycle in rats and produce insomnia symptom[32], which may be possible mechanism between insomnia and binge drinking of adolescents.

Consistent with previous studies, binge drinking was related to the use of cigarettes[33 34]. In our study, current smoking had a strongest effect on the odds of binge drinking, with boys and girls who smoked during the past 30 days having over 5 and 6 times higher possibility to binge drink. In addition, our result revealed that both fighting and being bullied were positively related with adolescent binge drinking, which highlight the need for adolescent violence prevention programs that focus on the reduction of alcohol abuse. We found positive association between binge drinking and sexual experiences, which was in correspondence with earlier study. Lewis et al. reported that alcohol facilitated formation of intimate relationship[35]. Our findings suggested that comprehensive and effective measure including raising alcohol prices[36], reducing alcohol outlet density[37], banning alcohol advertisement[38], should be taken to control binge drinking among adolescents in China.

Our study had several limitations. First, the study was based on a cross-sectional study, which did not allow us establish the temporal relationship between factors and binge drinking. Second, only school-going adolescents participated in the survey. Students who was expelled, suspended, or dropped out were excluded from the survey. If those were more likely to binge drinking, this may lead to underestimate the overall prevalence.

Acknowledgements The authors would like to thank all the students, parents, teachers and local officials for their participation, assistance and co-operation.

Contributor HW designed the study, and collected and analyzed the data with MY. JZ and RH were involved in data interpretation. HD took part in data analysis and revised the manuscript. MW was involved in data collection. All the authors have read and approved the final submitted version.

Competing interests None declared.

Funding The work was supported by grant (2016YFC0900502) from National Key Research and Development Program of China.

Ethics approval Ethics Committee of Zhejiang Provincial Centre for Disease Control and Prevention.

Data sharing statement No additional data are available

For peer review only - http://bmjopen.bmj.com/site/about/guidelines.xhtml

For peer review only

References

1. Rehm J, Mathers C, Popova S, et al. Global burden of disease and injury and economic cost attributable to alcohol use and alcohol-use disorders. *Lancet* 2009;373(9682):2223-33. doi: 10.1016/S0140-6736(09)60746-7
2. Collaborators GBDRF. Global, regional, and national comparative risk assessment of 84 behavioural, environmental and occupational, and metabolic risks or clusters of risks, 1990-2016: a systematic analysis for the Global Burden of Disease Study 2016. *Lancet* 2017;390(10100):1345-422. doi: 10.1016/S0140-6736(17)32366-8
3. Degenhardt L, O'Loughlin C, Swift W, et al. The persistence of adolescent binge drinking into adulthood: findings from a 15-year prospective cohort study. *BMJ Open* 2013;3(8):e003015. doi: 10.1136/bmjopen-2013-003015
4. Ehlers CL, Gizer IR, Vieten C, et al. Age at regular drinking, clinical course, and heritability of alcohol dependence in the San Francisco family study: a gender analysis. *Am J Addict* 2010;19(2):101-10. doi: 10.1111/j.1521-0391.2009.00021.x
5. Hicks BM, Iacono WG, McGue M. Consequences of an adolescent onset and persistent course of alcohol dependence in men: adolescent risk factors and adult outcomes. *Alcohol Clin Exp Res* 2010;34(5):819-33. doi: 10.1111/j.1530-0277.2010.01154.x
6. Hingson RW, Heeren T, Edwards EM. Age at drinking onset, alcohol dependence, and their relation to drug use and dependence, driving under the influence of drugs, and motor-vehicle crash involvement because of drugs. *J Stud Alcohol Drugs* 2008;69(2):192-201.
7. Viner RM, Taylor B. Adult outcomes of binge drinking in adolescence: findings from a UK national birth cohort. *J Epidemiol Community Health* 2007;61(10):902-7. doi: 10.1136/jech.2005.038117
8. Raposo J, Costa ACQ, Valenca PAM, et al. Binge drinking and illicit drug use among adolescent students. *Rev Saude Publica* 2017;51:83. doi: 10.11606/S1518-8787.2017051006863
9. Nkansah-Amankra S, Diedhiou A, Agbanu HL, et al. Correlates of sexual risk behaviors among high school students in Colorado: analysis and implications for school-based HIV/AIDS programs. *Matern Child Health J* 2011;15(6):730-41. doi: 10.1007/s10995-010-0634-3
10. Tavolacci MP, Boerg E, Richard L, et al. Prevalence of binge drinking and associated behaviours among 3286 college students in France. *BMC Public Health* 2016;16:178. doi: 10.1186/s12889-016-2863-x
11. The ESPAD Group: ESPAD Report 2015 Result from the European School Survey Project on Alcohol and Other Drugs. Lisbon, 2016.
12. Esser MB, Clayton H, Demissie Z, et al. Current and Binge Drinking Among High School Students - United States, 1991-2015. *MMWR Morb Mortal Wkly Rep* 2017;66(18):474-78. doi: 10.15585/mmwr.mm6618a4

For peer review only - http://bmjopen.bmj.com/site/about/guidelines.xhtml

13. Lu S, Du S, Hu X, et al. Drinking patterns and the association between socio-demographic factors and adolescents' alcohol use in three metropolises in China. *Int J Environ Res Public Health* 2015;12(2):2037-53. doi: 10.3390/ijerph120202037

14. Centers for Disease Control and Prevention . 2017 State and Local Youth Risk Behavior Survey; 2017 [Available from: https://www.cdc.gov/healthyyouth/data/yrbs/questionnaires.htm accessed 21/11 2017.

15. World Health Organization.Global school-based students health survey(GSHS) 2015 [updated 7/6. Available from: http://www.who.int/ncds/surveillance/gshs/nepal/en/ accessed 21/11 2017.

16. Pengpid S, Peltzer K. Alcohol use and associated factors among adolescent students in Thailand. *West Indian Med J* 2012;61(9):890-6.

17. Newsletter N. NIAAA Council Approves Definition of Binge Drinking. 2004.

18. Huang R, Ho SY, Wang MP, et al. Sociodemographic risk factors of alcohol drinking in Hong Kong adolescents. *J Epidemiol Community Health* 2016;70(4):374-9. doi: 10.1136/jech-2015-206418

19. Xing Y, Ji C, Zhang L. Relationship of binge drinking and other health-compromising behaviors among urban adolescents in China. *J Adolesc Health* 2006;39(4):495-500. doi: 10.1016/j.jadohealth.2006.03.014

20. Kwok KH, Yuan SN. Parental socioeconomic status and binge drinking in adolescents: A systematic review. *Am J Addict* 2016 doi: 10.1111/ajad.12461

21. Sokol-Katz J, Dunham R, Zimmerman R. Family structure versus parental attachment in controlling adolescent deviant behavior: a social control model. *Adolescence* 1997;32(125):199-215.

22. Mak KK, Ho SY, Thomas GN, et al. Family structure, parent-child conversation time and substance use among Chinese adolescents. *BMC Public Health* 2010;10:503. doi: 10.1186/1471-2458-10-503

23. van den Eijnden R, Mheen D, Vet R, et al. Alcohol-specific parenting and adolescents' alcohol-related problems: the interacting role of alcohol availability at home and parental rules. *J Stud Alcohol Drugs* 2011;72(3):408-17.

24. Alghadir AH, Gabr SA, Iqbal ZA. Television watching, diet and body mass index of school children in Saudi Arabia. *Pediatr Int* 2016;58(4):290-4. doi: 10.1111/ped.12834

25. Al-Ghamdi SH. The association between watching television and obesity in children of school-age in Saudi Arabia. *J Family Community Med* 2013;20(2):83-9. doi: 10.4103/2230-8229.114767

26. Mamun AA, O'Callaghan MJ, Williams G, et al. Television watching from adolescence to adulthood and its association with BMI, waist circumference, waist-to-hip ratio and obesity: a longitudinal study. *Public Health Nutr* 2013;16(1):54-64. doi: 10.1017/S1368980012002832

For peer review only - http://bmjopen.bmj.com/site/about/guidelines.xhtml

27. Mekary RA, Lucas M, Pan A, et al. Isotemporal substitution analysis for physical activity, television watching, and risk of depression. *Am J Epidemiol* 2013;178(3):474-83. doi: 10.1093/aje/kws590
28. Jiang XX, Hardy LL, Ding D, et al. Recreational screen-time among Chinese adolescents: a cross-sectional study. *J Epidemiol* 2014;24(5):397-403.
29. Page RM, Dennis M, Lindsay GB, et al. Psychosocial distress and substance use among adolescents in four countries: Philippines, China, Chile, and Namibia. *Youth & Society* 2011;43(3):900-30.
30. Stickley A, Koyanagi A, Koposov R, et al. Loneliness and health risk behaviours among Russian and U.S. adolescents: a cross-sectional study. *BMC Public Health* 2014;14:366. doi: 10.1186/1471-2458-14-366
31. Popovici I, French MT. Binge drinking and sleep problems among young adults. *Drug Alcohol Depend* 2013;132(1-2):207-15. doi: 10.1016/j.drugalcdep.2013.02.001
32. Sharma R, Bradshaw K, Sahota P, et al. Acute binge alcohol administration reverses sleep-wake cycle in Sprague Dawley rats. *Alcohol Clin Exp Res* 2014;38(7):1941-6. doi: 10.1111/acer.12463
33. Hale D, Viner R. Trends in the prevalence of multiple substance use in adolescents in England, 1998-2009. *J Public Health (Oxf)* 2013;35(3):367-74. doi: 10.1093/pubmed/fdt022
34. Stickley A, Koyanagi A, Koposov R, et al. Binge drinking among adolescents in Russia: prevalence, risk and protective factors. *Addict Behav* 2013;38(4):1988-95. doi: 10.1016/j.addbeh.2012.12.009
35. Lewis MA, Granato H, Blayney JA, et al. Predictors of hooking up sexual behaviors and emotional reactions among U.S. college students. *Arch Sex Behav* 2012;41(5):1219-29. doi: 10.1007/s10508-011-9817-2
36. Stockwell T, Zhao J, Martin G, et al. Minimum alcohol prices and outlet densities in British Columbia, Canada: estimated impacts on alcohol-attributable hospital admissions. *Am J Public Health* 2013;103(11):2014-20. doi: 10.2105/AJPH.2013.301289
37. Xuan Z, Blanchette J, Nelson TF, et al. The alcohol policy environment and policy subgroups as predictors of binge drinking measures among US adults. *Am J Public Health* 2015;105(4):816-22. doi: 10.2105/AJPH.2014.302112
38. Anderson P, Chisholm D, Fuhr DC. Effectiveness and cost-effectiveness of policies and programmes to reduce the harm caused by alcohol. *Lancet* 2009;373(9682):2234-46. doi: 10.1016/S0140-6736(09)60744-3

For peer review only

Table 1 questions comprising variables included in the survey

Variables	Questions and options
Parental education level	What is the highest level of education your father/mother has obtained? With six answer options(primary school or below, middle school, high school, college or university, master graduates or above, unknown)
Parental marital status	What is your parent s' current marital status?(Answer options: married, divorced, widowed, separated)
Siblings	Are you the only son/daughter of your parent?(Answer options: yes, no)
Physical activity	During the past 7 days, on how many days were you physically active for a total of at least 60 minutes per day?(Answer options: none,1 day, 2 day, 3 day, 4 day, 5 day, 6 day, 7 day)
Academic performance	How would you describe your grades in your class?(Excellent, middle, bad)
Screen time	On an average school day, how many hours do you play video or computer games or use a computer for something that is not school work? (Answer options: none, < 1 h/d, 1 h/d, 2 h/d, 3 h/d, 4 h/d, ≥5 h/d)
Loneliness	During the past 12 months, did you ever feel lonely? (Answer options: never, occasional, sometimes, often, always)
Insomnia	During the past 12 months, have you ever felt worried about something that you cannot fall asleep? (Answer options: never, occasional, sometimes, often, always)
Suicidal attempt	During the past 12 months, how many times did you actually attempt suicide? (Answer options: none,1 time,2-3times, 4-5 times,6 or more times)
Current smoking	During the past 30 days, on how many days did you smoke cigarettes? (Answer options:none,1 time,2-3times, 4-5 times,6 or more times)
Fighting	During the past 12 months, how many times were you in a physical fight? (Answer options: none,1 time,2-3times, 4-5 times, 6-7 times,8-9 times,10-11 times,12 or more times)
Being bullied	During the past 12 months, how many times has someone threatened or injured you with a weapon such as a gun, knife, or club on school property? (Answer options: none,1 time,2-3times, 4-5 times, 6-7 times,8-9 times,10-11 times,12 or more times)
Sexual experience	Have you ever had sexual intercourse? (Answer options: yes no)

For peer review only - http://bmjopen.bmj.com/site/about/guidelines.xhtml

Table 2 weighted prevalence of current drinking and binge drinking by different characteristics

Characteristics	Current drinking			Binge drinking*		
	Prevalence (%)*	Rao-Scott Chi-Square	*P*	Prevalence (%)*	Rao-Scott Chi-Square	*P*
Sex		93.65	<.0001		80.46	<.0001
Boys	27.0 (25.6-28.5)			11.8 (10.8-12.9)		
Girls	18.2(16.8-19.6)			6.5(5.6-7.3)		
Area		2.38	0.12		0.62	0.4314
Urban	24.4 (21.9-26.9)			9.8 (8.2-11.4)		
Rural	22.0 (20.5-23.5)			9.0 (8.0-10.0)		
Types of school		319.93	<.0001		278.29	<.0001
Middle school	17.5 (16.2-18.8)			6.3 (5.6-7.0)		
Academic high school	22.1 (20.4-23.8)			7.7 (6.8-8.7)		
Vocational high school	35.9 (33.7-38.0)			17.9 (15.9-20.0)		

*Based on the weighted data.

For peer review only

For peer review only - http://bmjopen.bmj.com/site/about/guidelines.xhtml

Table 3 socio-demographic and characteristics of adolescents from Zhejiang (N=23543)

Characteristics	Boys	Girls	Chi-square	*P*
Age range (years)			1.98	0.5771
≤13	2689(22.3)	2470(21.5)		
14	2192(18.2)	2108(18.4)		
15	1905(15.7)	1825(15.9)		
≥16	5282(43.8)	5072(44.2)		
Area			4.68	0.0306
Urban	4544(37.7)	4478(39.0)		
Rural	7524(62.3)	6997(61.0)		
Types of school			9.63	0.008
Middle school	6364(52.7)	5843(50.9)		
Academic high school	3223(26.7)	3254(28.4)		
Vocational high school	2481(20.6)	2378(20.7)		
Parental marital status			12.56	0.0004
Married	10924(90.5)	10227(89.1)		
Others	1144(9.5)	1248(10.9)		
Paternal education level			10.68	0.0136
Primary or below	6908(57.2)	6660(58.1)		
Middle or high school	2628(21.8)	2472(21.5)		
College or above	1575(13.1)	1554(13.5)		
Unknown	957(7.9)	789(6.9)		
Maternal education level			44.34	<.0001
Primary or below	7292(60.4)	7238(63.1)		
Middle or high school	2271(18.8)	2092(18.2)		
College or above	1392(11.6)	1344(11.7)		
Unknown	1113(9.2)	801(7.0)		
Having siblings			625.18	<.0001
Yes	5263(43.6)	6874(59.9)		
No	6805(56.4)	4601(40.1)		
Academic performance			118.53	<.0001
Excellent	2731(22.6)	2717(23.7)		
Middle	5727(47.5)	6038(52.6)		
Bad	3610(29.9)	2720(23.7)		
Physical activity (d/w)			411.53	<.0001
0	2079(17.2)	2804(24.4)		
1-2	2703(22.4)	2987(26.0)		
3-5	4237(35.1)	3813(33.2)		
6-7	3049(25.3)	1871(16.3)		
Screen time (h/d)			72.36	<.0001
0	3604(29.9)	3651(31.8)		
<1	1968(16.3)	2041(17.8)		
1-4	3853(31.9)	3768(32.8)		
≥4	2643(21.9)	2015(17.6)		
Loneliness			84.27	<.0001
Never/occasional	8082(67.0)	7040(61.4)		
Sometimes	2698(22.4)	3085(26.9)		
Often/always	1288(10.6)	1350(11.7)		
Insomnia			103.91	<.0001
Never/occasional	9880(81.9)	8802(76.7)		
Sometimes	1653(13.7)	2112(18.4)		
Often/always	535(4.4)	561(4.9)		
Suicide attempt			49.37	<.0001
Yes	337(2.79)	517(4.51)		
No	11731(97.21)	10958(95.49)		

For peer review only - http://bmjopen.bmj.com/site/about/guidelines.xhtml

Current drinking			366.40	<.0001
Yes	968(8.0)	279(2.4)		
No	11100(92.0)	11196(97.6)		
Fighting			1455.74	<.0001
Yes	2966(24.6)	741(6.5)		
No	9102(75.4)	10734(93.5)		
Being bullied			148.26	<.0001
Yes	1872(15.5)	1169(10.2)		
No	10196(84.5)	10306(89.8)		
Sexual experience			108.07	<.0001
Yes	591(4.9)	270(2.4)		
No	11477(95.1)	11205(97.6)		

For peer review only

For peer review only - http://bmjopen.bmj.com/site/about/guidelines.xhtml

Table 4 crude and adjusted odd ratio of factors associated with binge drinking among adolescents in China

Variable	Boys (N=12 068)		Girls (N=11 475)	
	COR(95%CI)	AOR(95%CI)	COR(95%CI)	AOR(95%CI)
Age groups(ref: ≤13 years)				
14	**1.95 (1.52-2.50) &**	**1.96 (1.51-2.54) &**	1.12 (0.77-1.65)	1.16 (0.75-1.78)
15	**2.70(2.06-3.53) &**	**2.19(1.66-2.90) &**	**1.46(1.01-2.12) #**	1.14(0.75-1.75)
≥16	**3.89(3.07-4.92) &**	**2.10(1.55-2.84) &**	**1.68(1.13-2.50) ***	1.06(0.61-1.85)
Rural(ref: Urban)	1.00(0.77-1.29)	1.07(0.89-1.30)	0.77(0.57-1.04)	0.89(0.72-1.11)
Types of school (ref: Middle school)				
Academic high school	**1.62(1.33-1.96) &**	**1.48(1.15-1.90) ***	0.78(0.60-1.00)	0.97(0.61-1.55)
Vocational high school	**3.65(2.91-4.58) &**	**2.12(1.62-2.78) &**	**2.71(2.07-3.55) &**	**2.11(1.41-3.17) $**
Parental marital status(ref: Married)				
Others	**1.32(1.08-1.61) ***	0.92(0.74-1.16)	**2.07(1.67-2.57) &**	1.23(0.93-1.21)
Paternal education level				
(ref: Primary or below)				
Middle or high school	1.00(0.84-1.20)	1.11(0.90-1.37)	0.84(0.68-1.05)	0.94(0.73-1.21)
College or above	**0.76(0.60-0.95) #**	0.92(0.68-1.24)	**0.66(0.49-0.90) ***	0.78(0.51-1.19)
Unknown	0.91(0.72-1.16)	1.25(0.93-1.68)	1.23(0.89-1.71)	1.43(0.87-2.34)
Maternal education level				
(ref: Primary or below)				
Middle or high school	0.99(0.84-1.17)	1.06(0.86-1.31)	0.99(0.77-1.27)	1.18(0.90-1.53)
College or above	0.78(0.61-1.00)	1.06(0.76-1.48)	0.77(0.56-1.05)	1.07(0.67-1.69)
Unknown	0.82(0.64-1.06)	0.72(0.52-1.01)	1.11(0.77-1.61)	0.79(0.48-1.30)
Siblings(ref: No)	0.92(0.80-1.06)	0.98(0.85-1.13)	0.99(0.80-1.22)	1.05(0.84-1.29)
Academic performance(ref: Excellent)				
Middle	**1.23(1.01-1.50) #**	1.13(0.93-1.37)	1.28(0.95-1.73)	1.19(0.88-1.60)
Bad	**1.82(1.49-2.22)** &	**1.27(1.04-1.56) #**	**1.97(1.43-2.70)** &	1.32(0.96-1.81)
Physical activity (ref: 0 d/w)				
1-2 d/w	0.82(0.66-1.02)	0.90(0.70-1.16)	0.86(0.70-1.06)	1.09(0.88-1.37)
3-5 d/w	**0.79(0.65-0.96) #**	0.92(0.75-1.14)	0.78(0.60-1.02)	1.02(0.78-1.35)
6-7 d/w	1.09(0.90-1.33)	**1.33(1.08-1.66) #**	0.95(0.74-1.21)	1.21(0.90-1.63)
Screen time (ref: 0 h/d)				
<1 h/d	0.90(0.72-1.12)	0.94(0.75-1.17)	1.27(0.88-1.84)	1.17(0.80-1.71)
1-4 h/d	**1.47(1.19-1.82) $**	1.14(0.91-1.43)	**2.13(1.62-2.80) &**	**1.59(1.18-2.14) ***
≥4 h/d	**2.79(2.28-3.41) &**	**1.45(1.17-1.80) $**	**5.91(4.53-7.71) &**	**2.59(1.92-3.49) &**
Loneliness(ref: Never/Occasionally)				
Sometimes	**1.42(1.23-1.63) &**	1.08(0.91-1.28)	**1.69(1.37-2.08) &**	1.23(0.96-1.59)

For peer review only

For peer review only - http://bmjopen.bmj.com/site/about/guidelines.xhtml

Often/Always	**2.25(1.87-2.72) &**	1.17(0.89-1.55)	**3.14(2.51-3.93) &**	**1.44(1.05-1.96) #**
Insomnia(ref: Never/Occasionally)				
Sometimes	**1.97(1.68-2.32) &**	**1.41(1.13-1.77) ***	**1.92(1.59-2.31) &**	**1.33(1.06-1.66) $**
Often/Always	**2.89(2.29-3.64) &**	**1.68(1.23-2.30) ***	**5.18(4.05-6.62) &**	**2.22(1.62-3.04) &**
Suicide attempt(ref: No)	**4.14(3.24-5.30) &**	**2.37(1.73-3.24) &**	**7.35(5.51-9.81) &**	**3.36(2.48-4.57) &**
Current smoking(ref: No)	**11.24(9.13-13.84) &**	**5.21(4.14-6.56) &**	**20.27(15.32-26.83) &**	**6.81(4.69-9.90) &**
Fighting(ref: No)	**2.78(2.37-3.27) &**	**2.18(1.81-2.62) &**	**5.07(4.04-6.37) &**	**2.47(1.86-3.28) &**
Being bullied(ref: No)	**1.59(1.34-1.89) &**	**1.23(1.03-1.46) #**	**1.81(1.27-2.59) ***	1.05(0.70-1.57)
Sexual experience(ref: No)	**6.92(5.33-8.98) &**	**2.32(1.68-3.19) &**	**6.60(4.94-8.83) &**	**1.82(1.21-2.73) ***

Bold numbers represent significant results

COR: Crude odds ratio. AOR: Adjusted odds ratios. CI: confidence intervals. #: $P<0.05$. *: $P<0.01$. $: $P<0.001$. &: $P<0.0001$

AOR is adjusted for all other covariates in the model

For peer review only

STROBE Statement—checklist of items that should be included in reports of observational studies

	Item No	Recommendation
**Title and abstract**	1 **YES Title:page1 Abstract: page2**	(*a*) Indicate the study's design with a commonly used term in the title or the abstract
		(*b*) Provide in the abstract an informative and balanced summary of what was done and what was found
**Introduction**		
Background/rationale	2 **YES Page3-4**	Explain the scientific background and rationale for the investigation being reported
Objectives	3 **YES Page 3**	State specific objectives, including any prespecified hypotheses
**Methods**		
Study design	4 **YES Page3-4**	Present key elements of study design early in the paper
Setting	5 **YES Page3-4**	Describe the setting, locations, and relevant dates, including periods of recruitment, exposure, follow-up, and data collection
Participants	6 **YES Page3-4**	(*a*) *Cohort study*—Give the eligibility criteria, and the sources and methods of selection of participants. Describe methods of follow-up<br>*Case-control study*—Give the eligibility criteria, and the sources and methods of case ascertainment and control selection. Give the rationale for the choice of cases and controls<br>*Cross-sectional study*—Give the eligibility criteria, and the sources and methods of selection of participants
		(*b*) *Cohort study*—For matched studies, give matching criteria and number of exposed and unexposed<br>*Case-control study*—For matched studies, give matching criteria and the number of controls per case
Variables	7 **YES Page11**	Clearly define all outcomes, exposures, predictors, potential confounders, and effect modifiers. Give diagnostic criteria, if applicable
Data sources/ measurement	8* **YES Page 4，11**	For each variable of interest, give sources of data and details of methods of assessment (measurement). Describe comparability of assessment methods if there is more than one group
Bias	9 **YES Page 4**	Describe any efforts to address potential sources of bias
Study size	10 **YES Page3-5**	Explain how the study size was arrived at
Quantitative variables	11 **YES**	Explain how quantitative variables were handled in the analyses. If applicable, describe which groupings were chosen and why
Statistical methods	12 **YES Page 4**	(*a*) Describe all statistical methods, including those used to control for confounding
		(*b*) Describe any methods used to examine subgroups and interactions
		(*c*) Explain how missing data were addressed
		(*d*) *Cohort study*—If applicable, explain how loss to follow-up was addressed<br>*Case-control study*—If applicable, explain how matching of cases and controls was addressed

For peer review only

		Cross-sectional study—If applicable, describe analytical methods taking account of sampling strategy
		(*e*) Describe any sensitivity analyses
**Results**		
Participants	13* **YES Page5**	(a) Report numbers of individuals at each stage of study—eg numbers potentially eligible, examined for eligibility, confirmed eligible, included in the study, completing follow-up, and analysed
		(b) Give reasons for non-participation at each stage
		(c) Consider use of a flow diagram
Descriptive data	14* **YES Page 13**	(a) Give characteristics of study participants (eg demographic, clinical, social) and information on exposures and potential confounders
		(b) Indicate number of participants with missing data for each variable of interest
		(c) *Cohort study*—Summarise follow-up time (eg, average and total amount)
Outcome data	15* **YES Page 12,14**	*Cohort study*—Report numbers of outcome events or summary measures over time
		Case-control study—Report numbers in each exposure category, or summary measures of exposure
		Cross-sectional study—Report numbers of outcome events or summary measures
Main results	16 **YES Page14**	(*a*) Give unadjusted estimates and, if applicable, confounder-adjusted estimates and their precision (eg, 95% confidence interval). Make clear which confounders were adjusted for and why they were included
		(*b*) Report category boundaries when continuous variables were categorized
		(*c*) If relevant, consider translating estimates of relative risk into absolute risk for a meaningful time period
Other analyses	17 **No**	Report other analyses done—eg analyses of subgroups and interactions, and sensitivity analyses
**Discussion**		
Key results	18 **YES Page 5**	Summarise key results with reference to study objectives
Limitations	19 **YES Page 7**	Discuss limitations of the study, taking into account sources of potential bias or imprecision. Discuss both direction and magnitude of any potential bias
Interpretation	20 **YES page 6-7**	Give a cautious overall interpretation of results considering objectives, limitations, multiplicity of analyses, results from similar studies, and other relevant evidence
Generalisability	21 **YES Page7**	Discuss the generalisability (external validity) of the study results
**Other information**		
Funding	22 **YES Page 8**	Give the source of funding and the role of the funders for the present study and, if applicable, for the original study on which the present article is based

*Give information separately for cases and controls in case-control studies and, if applicable, for exposed and unexposed groups in cohort and cross-sectional studies.

For peer review only

For peer review only - http://bmjopen.bmj.com/site/about/guidelines.xhtml

Note: An Explanation and Elaboration article discusses each checklist item and gives methodological background and published examples of transparent reporting. The STROBE checklist is best used in conjunction with this article (freely available on the Web sites of PLoS Medicine at http://www.plosmedicine.org/, Annals of Internal Medicine at http://www.annals.org/, and Epidemiology at http://www.epidem.com/). Information on the STROBE Initiative is available at www.strobe-statement.org.

For peer review only

For peer review only - http://bmjopen.bmj.com/site/about/guidelines.xhtml

BMJ Open

Binge Drinking and Associated Factors among School Students: A Cross-sectional Study in Zhejiang Province, China

Journal:	*BMJ Open*
Manuscript ID	bmjopen-2017-021077.R1
Article Type:	Research
Date Submitted by the Author:	25-Jan-2018
Complete List of Authors:	Wang, Hao; Zhejiang Provincial Center for Diseases Control and Prevention, Department of NCDs Control and Prevention<br>Hu, Ru-Ying; Zhejiang Provincial Center for Diseases Control and Prevention, Department of NCDs Control and Prevention<br>Zhong, Jie-Ming; Zhejiang Provincial Center for Diseases Control and Prevention, Department of NCDs Control and Prevention<br>Du, Huaidong; University of Oxford, Clinical Trial Service Unit & Epidemiological Studies Unit (CTSU), Nuffield Department of Population Health; University of Oxford, Medical Research Council Population Health Research Unit, Nuffield Department of Population Health<br>Wang, Meng; Zhejiang Provincal Center for Disease Control and Prevention, Department of NCDs Control and Prevention<br>Yu, Min; Zhejiang Provincial Center for Diseases Control and Prevention
**Primary Subject Heading**:	Public health
Secondary Subject Heading:	Public health
Keywords:	Binge drinking, Alcohol, Adolescents, Factors

SCHOLARONE™
Manuscripts

Binge Drinking and Associated Factors among School Students: A Cross-sectional Study in Zhejiang Province, China

Hao Wang[1], Ruying Hu[1], Jieming Zhong[1], Huaidong Du[2, 3], Wang Meng[1], Min Yu[1]

Author affiliations

1. Department of NCDs Control and Prevention, Zhejiang Pcial Center for Diseases Control and Prevention, Hangzhou, China

2. Clinical Trial Service Unit & Epidemiological Studies Unit (CTSU), Nuffield Department of Population Health, University of Oxford, Oxford, UK

3. Medical Research Council Population Health Research Unit, Nuffield Department of Population Health, University of Oxford, Oxford, UK

Correspondence to Min Yu; myu@cdc.zj.cn

For peer review only

Abstract

Objective: To investigate the prevalence and correlating factors of binge drinking among middle and high school students in Zhejiang Province, China.

Methods: We performed a cross-sectional study using data from a school-based survey. During the survey, a total of 23 543 (response rate =97.5%) eligible adolescents from 442 different schools (including middle schools, academic high schools and vocational high schools) were asked to fill in an anonymous self-administered behaviour questionnaire between April and May 2017. Multivariable logistic regression models were used for data analyses.

Results: The mean (SD) age of the participants was 15.6 years (1.7) and 51.3% of them were boys. The proportion of students of middle schools, academic high schools and vocational high schools were 51.9%, 27.5% and 20.6%, respectively. Totally, 22.8% (95%CI: 21.6-23.9) of students reported drinking alcohol in the past 30 days and 9.2% (95%CI: 8.5-10.0) of students reported binge drinking (i.e., drinking four or more alcoholic drinks for girl in a row of 1 to 2 hours and five or more for boy) during the past month. In comparison with students of middle school (6.3%) and academic high school (7.7%), the binge drinking prevalence among vocational high school students was the highest (17.9%). Older age, high school students, poor academic performance, more physical activity, excessive exposure to electronic screen products, loneliness, insomnia, suicide attempt, smoking, fighting, being bullied, sexual experience were found to be positively associated with adolescent binge drinking.

Conclusions: Binge drinking is common among middle and high school students in Zhejiang, China. Efforts to prevent binge drinking may need to address a cluster of socio-demographic and behavioural factors. Our findings would be instructive for healthcare providers to detect students with high-risk of binge drinking, which will aid in planning intervention measures for at-risk students.

Keywords: Binge drinking; Alcohol; Adolescents; Factors

For peer review only - http://bmjopen.bmj.com/site/about/guidelines.xhtml

Strengths and limitations of this study

- This is a school-based study with a relatively large sample size, a high response rate and a representative Provincial sample from China.
- The study questionnaire includes a great number of factors, which might help us better understand how to prevent adolescent binge drinking.
- This is a cross-sectional study, which do not allow us establish the causal relationship between correlating factors and binge drinking.
- Only students attending schools participated in the survey. Given the possibility that students being expelled, suspended, or dropped out from schools may be more likely to binge drink, the overall prevalence of binge drinking observed in our current study might represent a conservative underestimation of the situation.

For peer review only

Introduction

Alcohol consumption has been identified as an important risk factor for chronic disease and injury [1]. DALYs (disability-adjusted life years) attributable to alcohol consumption have increased by more than 25% over the years 1990 – 2016, accounting for an estimated 99.2 million worldwide. In 2016, alcohol consumption is responsible for more than 2.8 million deaths worldwide, including approximately 0.7 million in China[2]. Many studies have clearly demonstrated that alcohol misuse of adolescents was associated with motor vehicle collisions[3], violence and crime[4 5], mental health and suicidal behaviours[6-8]. Moreover, alcohol use during early adolescence is a risk factor for the later development of alcohol dependence[9-11].

Binge drinking is defined as episodic excessive drinking. Prevalence of binge drinking among adolescents varied across different countries, with 8% in Iceland and 56% in Denmark[12]. Among 25 European countries, prevalence of binge drinking among adolescents rose from 36% in 1995 to 42% in 2007, and then declined to 35% in 2015[12]. In the United States, the corresponding figure declined from 31.3% in 1991 to 17.7% in 2015[13]. In Asia, the proportion of Heavy episodic drinking (i.e., binge drinking) of grades 7-12 students was 10.3% in Korea[14], 21.2% of students with mean age 14.7 years reported to drink alcohol in the past 30 days, and 7.1% of students reported binge drinking in Hong Kong[15]. In mainland China, a study of 7344 middle school students conducted in 4 cities (Beijing, Hangzhou, Wuhan, Urumchi) found that prevalence of current drinking was 14.4%, and 9.9% of students had experienced drunkenness, and significant regional difference was found in all the cities[16]. Another study conducted in 2004 including 54 040 students in grades 7-12 from 18 Provincial capitals in China found that during the past 30 days from the survey time, 25.2% students reported consuming at least one alcoholic drink and 10.3% reported at least one episode of binge drinking[17]. An up-to-date study, surveyed 13 811 high school students from China's 3 metropolises (Beijing, Shanghai, and Guangzhou) in 2013, found the prevalence of binge drinking was 4.8%[18]. Due to some reason, not all of the grades were covered, yielding an underestimation of prevalence.

Parental alcohol-related practices were important risk factors of adolescent drinking[19]. In traditional Chinese culture, alcohol drinking is both a normal part of the daily diet, especially in rural areas, and an important part of rituals, business occasions, festivals, and special events. Moderate drinking on important occasions is encouraged for adults[20]. A small amount or sips of alcoholic beverage may be allowed for children by parents, especially grandfathers[18]. Some previous surveys have found that nearly half of senior high schools students in China have drunk at home with their parents or out with their peers[21 22].

Previous studies have found some inconsistent socio-demographic difference of adolescent binge drinking. For instance, Miller et al. reported that binge drinking prevalence was similar among high school boys and girls in the United States[23], while boys in Hong Kong had higher prevalence of binge drinking than girls[15]. Sokol-Katz et al. reported that family structure was not

For peer review only - http://bmjopen.bmj.com/site/about/guidelines.xhtml

significantly related to any of the deviant behaviours (such as alcohol, cigarette, and drug use) in middle school students in Florida, after controlling for gender and race/ethnicity[24], while non-intact family was associated with substance use among Chinese adolescents in Hong Kong[25].

Zhejiang Province, standing in the east of China, has a population of 56 million. It has experienced rapid economic development in the past 30 years. With the economic development and globalization, adolescents living in this area are more likely to be influenced by western culture. Therefore, the patterns of alcohol use in this area might differ from those in other regions of China. The current study was designed with an aim to examine the prevalence of binge drinking and ascertain its correlating factors among students in Zhejiang Province.

Methods

Survey design and participants

The survey utilized a three-stage sampling design. In stage one, 30 counties, including 12 urban areas and 18 rural areas, were sampled randomly from all 90 counties (31 urban and 59 rural) of Zhejiang Province according to socioeconomic status. In stage two, 10 classes of middle school, 5 classes of academic high school, and 5 classes of vocational high school were selected randomly within each chosen counties, respectively. In stage three, all of the students in the chosen classes were invited to participate our study. In China, after 6-years education at primary schools, children usually attend middle schools (i.e., junior high school) for 3-years education (grades 7-9). After graduation from middle schools, they enter high schools (i.e., senior high schools, including academic high schools and vocational high schools) for another 3-years education (grades 10-12). The survey questionnaire was modelled from existing surveys including Youth Risk Behaviour Survey (YRBS) conducted by the Centers for Disease Control and Prevention (CDC)[26] and the international Global School-based Student Health Survey supported by the World Health Organization[27]. Survey questions addressed demographic characteristics (including age, gender, parental educational level, parental marital status, number of siblings etc.), tobacco and alcohol use, physical activity, violence, injuries, suicidal behaviours, sexual behaviours. The survey was conducted between April and May 2017. Participants filled in the anonymous self-administrated questionnaire in the classrooms. The finished questionnaires were handed in on the spot.

Sample size calculation

The sample size was calculated by using the formula: $N=deff \times \mu^2 \times P \times (1-P)/d^2$. Means and 95% confidence interval (CI; 2-sided for u=1.96) were determined; the prevalence of binge drinking (10%) obtained in the China was used as a measure of probability (p)[17]; the design effect ($deff$) value was set at 3; and the relative error was: $d=r\times1\%$, r=15%. Based on these parameters, the sample size for each stratum was estimated to be 4610 subjects. Because there were 4 strata

For peer review only - http://bmjopen.bmj.com/site/about/guidelines.xhtml

(Areas: urban and rural. Sex: boy and girl), and assuming a potential nonresponse rate of 20%, the final sample size was calculated as 23 050.

Measures

Outcome variables

Alcoholic drinks include beer, wine, wine coolers, rice wine, and liquor such as Chinese liquor, rum, gin, vodka, or whiskey. Current drinking was assessed through the question: “During the past 30 days, on how many days did you have at least one drink of alcohol? (Options include: “0 days”, “1-2 days”, “3-5 days”, “6-9 days”, “10-19 days”, “20-29 days”, and “30 days”)”. Participants were considered as current drinkers if they answered had drunk at least 1-2 days during the past 30 days. Binge drinking was assessed by the question: “During the past 30 days, on how many days did you have 4 or more drinks of alcohol in a row (if you are girl) or 5 or more drinks of alcohol in a row of 1 to 2 hours (if you are boy)?[28] (Options include: “0 days”, “1 day”, “2 days”, “3-5 days”, “6-9 days”, “10-19 days”, “≥20 days”).” Participants were defined as binge drinkers if they answered at least 1 day.

Main covariates

Physical activity was assessed by the question: “During the past 7 days, on how many days were you physically active for a total of at least 60 minutes per day?”. Answer options included: “None”, “1 day”, “2 days”, “3 days”, “4 days”, “5 days”, “6 days” and “7 days”. Answers were further categorized into four groups: “None”, “1-2 d/w”, “3-5 d/w”, and “6-7 d/w”. Current smoking was assessed by the question: “During the past 30 days, on how many days did you smoke cigarettes?”. Answer options included: “None”, “1-2 days”, “3-5 days”, “6-9 days”, “10-19 days”, “20-29 days” and “all 30 days”. Current smoking was defined as smoking cigarettes at least on one day in the past 30 days. Screen time was collected through the question: “On an average school day, how many hours do you play video or computer games or use a computer for something that is not school work?”. Answer options included: “None”, “< 1 h/d”, “1 h/d”, “2 h/d”, “3 h/d”, “4 h/d”, “≥ 5 h/d”). Answers were further categorized into four groups: “None”, “<1 h/d”, “1-4 h/d”, “≥ 4 h/d”. Suicidal attempt was assessed using the question: “During the past 12 months, how many times did you actually attempt suicide?”. Response options included: “None”, “1 time”, “2-3 times”, “4-5 times”, “6 or more times”. Suicide attempt was defined as attempting suicide at least one time in the past 12 months. Fighting was assessed by the question: “During the past 12 months, how many times were you in a physical fight?”. Answer options included “None”, “1 time”, “2-3 times”, “4-5 times”, “6-7 times”, “8-9 times”, “10-11 times”, “12 or more times”. Fighting was defined physical fight at least one time in the past 12 months. Being bullied was assessed by the question: “During the past 12 months, how many times has someone threatened or injured you with a weapon such as a gun, knife, or club on school property?”.

For peer review only - http://bmjopen.bmj.com/site/about/guidelines.xhtml

For peer review only

Answer options included “None”, “1 time”, “2-3 times”, “4-5 times”, “6-7 times”, “8-9 times”, “10-11 times”, “12 or more times”. Being bullied was defined as being threatened or injured by someone at least one time in the past 12 months. Answer was dichotomized into “Yes” and “No”. More detailed covariates information was provided in the **Table 1**.

Quality Control

The survey was conducted by local Center for Disease Control and Prevention. All of the surveyors were trained before the survey. In order to improve response rate, every recruited student was given a pencil box as a gift. The survey was anonymous, and without filling in the participants' name on the questionnaire.

Ethics Statement

The study design and procedure was approved by the ethics committee of Zhejiang Provincial Centre for Disease Control and Prevention. Written informed consent was obtained from all participants and their guardians before survey.

Statistical analysis

All analyses were performed with SAS software V.9.3. A weighting factor was applied to each student record to adjust for non-response and for the varying probabilities of selection. The weight used for estimation in this survey is given by: $W = W1 \times W2 \times f1 \times f2$. W1 = the inverse of the probability of selecting the county. W2 = the inverse of the probability of selecting the classroom within the county. f1 = a student-level nonresponse adjustment factor calculated by class. f2 = a post-stratification adjustment factor calculated by grade[29]. Continuous variables were given as the mean ± standard deviation. The prevalence of current drinking and binge drinking was given as percent and 95% confidence intervals (CI). Weighted prevalence was calculated using the PROC SURVEYFREQ procedure and its difference between groups was compared using Rao-Scott Chi-square test. To assess the associations between each correlating factor and binge drinking, univariate and multivariable logistic regression analyses were performed using the PROC SURVEYLOGISTC procedure, to take into account the complex survey sampling methods. We first determine which factors were associated with binge drinking in univariate analyses ($P<0.05$). Variables significant in the univariate analyses were entered in a multivariable logistic regression model in a next step. All statistical tests were two tailed, and *P*-values <0.05 were considered statistically significant.

Results

Descriptive statistics

For peer review only - http://bmjopen.bmj.com/site/about/guidelines.xhtml

A total of 24 157 students was in the chosen classes and due to missing or incompletion of questionnaires and refusal to participation, 23 543 eligible participants (response rate 97.5%) were in the current analysis. Of which, 12 068 (51.3%) were boys and the average age was 15.6 years for both boys and girls. 12 207 (51.9%) of the participants were middle school students, 6 477 (27.5%) were academic high school students and 4 859 (20.6%) came from vocational high schools.

Table 2 demonstrated that 9.9% of students came from non-intact families. 11.4% of students' paternal educational level was college or above. 17.7% of students' maternal educational level was middle or high school. 54.8% of students had siblings. 22.5% of students reported having excellent academic performance. 20.7% of students reported being physically active 6-7 days per week. 21.5% of students reported spending more than 4 hours screen time per day. 64.1% of students reported never or occasionally feeling lonely in the past 12 months. 4.7% of students reported being often or always worried about something and cannot fall asleep in the past 12 months. 5.5% of students reported smoking cigarettes in the past 30 days. 15.6% of students reported engaging in physical fight in the past 12 months. 13.2% reported being bullied in the past 12 months. 3.9% of students reported ever having sexual experience.

The prevalence of current drinking and binge drinking

As shown in the **Table 3**, the prevalence of current drinking was 22.8% (95%CI: 21.6-23.9), higher in boys than in girls (27.0% vs. 18.2%), higher in students who ever attempted suicide (48.4% vs. 21.8% in those without suicide attempt), current smokers (73.3% vs. 19.8% in non-smokers), ever engaged in physical fight (40.3% vs. 19.5% in those without physical fight), ever being bullied (31.1% vs. 21.5% in those without being bullied), and those having sexual experience (59.1% vs. 21.3% in those without sexual experience). There was no statistical significance between urban and rural areas (24.4% vs. 22.0%). In addition, the prevalence of current drinking increased with poorer academic performance and the increase of screen time, severity of loneliness, and severity of insomnia (all P for trend <0.0001).

The prevalence of binge drinking was 9.2% (95%CI: 8.5-10.0) overall, and was 6.3%, 7.7% and 17.9%, respectively in middle school, academic high school, and vocational high school. Binge drinking was similarly associated with gender (11.8% in boys vs. 6.5% in girls), suicidal behaviour (31.5% vs. 8.4%), smoking (51.2% vs. 6.8%), physical fight (21.5% vs. 7.0%), being bullied (14.1% vs. 8.5%), sexual experience (39.2% vs. 8.1%), academic performance, screen time, severity of loneliness, and severity of insomnia (P for trend <0.0001 for the latter 4 parameters). No urban-rural difference in binge drinking was found either (9.8% vs. 9.0%).

Logistic regression analysis

For peer review only - http://bmjopen.bmj.com/site/about/guidelines.xhtml

After adjusting for other variables in the model, multivariable analysis showed that compared to boys aged ≤13 years, boys with older age were more likely to binge drink (**Table 4**). Compared to boys in middle school, boys of academic high school and vocational high school had 1.5 (OR=1.48, 95%CI: 1.15-1.90) and 2.1 (OR=2.09, 95%CI: 1.59-2.74) times more likely probability of binge drinking. Girls of vocational high school were 2.2 times more likelihood of binge drinking in comparison to girls of middle school (OR=2.18, 95%CI: 1.49-3.19). Boys with poor academic performance were 1.3 times more likely to binge drink in comparison to boys with excellent academic performance (OR=1.27, 95%CI: 1.03-1.56). Compared to boys who were not physically active within the past 7 days, boys being physically active on 6-7 days had a higher risk of binge drinking (OR=1.33, 95%CI: 1.07-1.66). Compared to girls who spent no screen-time, girls who spent 1-4 hours per day and more than 4 hours per day were 1.6 times (OR=1.61, 95%CI: 1.20-2.17) and 2.6 (OR=2.59, 95%CI: 1.93-3.48) times more likelihood of binge drinking. Boys who spent more than 4 hours per day on electronic screen products had a higher risk of binge drinking in comparison to boys who spent no time on such products (OR=1.33, 95%CI: 1.07-1.66). Girls who often or always felt lonely were 1.4 times more likely to binge drink than girls who never or occasionally felt lonely (OR=1.43, 95%CI: 1.05-1.95). Boys ever attempted suicide had a 2.4 times increased odds of binge drinking than boys without committing suicide (OR=2.37, 95%CI: 1.73-3.25). The corresponding odds ratio of girls was 3.4 (OR=3.36, 95%CI: 2.48-4.56). Both boys and girls who smoked cigarettes within the past 30 days were 5.2 times (OR=5.21, 95%CI: 4.13-6.58) and 6.7 (OR=6.68, 95%CI: 4.64-9.60) times more likely to binge drink than their counterparts who did not smoke cigarettes. Boys ever involved in physical fight in past 12 months had a 2.2 times higher risk of binge drinking than those not in physical fights (OR=2.18, 95%CI: 1.73-3.25). The corresponding figure of girls was 2.5 (OR=2.48, 95%CI: 1.87-3.29). Boys ever being bullied within the past 12 months had a 1.2 times higher risk of binge drinking than boys not being bullied (OR=1.23, 95%CI: 1.03-1.46). Both boys and girls who had sexual experience were 2.3 times (OR=2.33, 95%CI: 1.69-3.20) and 1.8 (OR=1.82, 95%CI: 1.22-2.72) times more likely to binge drink than their counterparts without sexual experience.

Discussion

In this study of middle and high school students in Zhejiang, China, we examined the prevalence of current drinking and binge drinking, identified socio-demographic and behavioural correlates of binge drinking, quantified the strength of associations, and provided information for preventive and intervention measures of binge drinking.

Prevalence of binge drinking

Due to different definition of binge drinking, it is difficult to compare different studies directly. Since using a 5-drink measure for high school students in 1975, most national surveys defined binge drinking as 5 or more drinks for both women and men[12 17]. A new gender-specific measure

For peer review only - http://bmjopen.bmj.com/site/about/guidelines.xhtml

of ≥ 4/5 drinks for women/wen used by Harvard School, because of gender differences in the risk of alcohol-related harms at these levels[30-32]. The use of new threshold was also justified because women generally have a smaller stature than men and because of physiological difference that affect the absorption and distribution of alcohol[33]. Besides, the definition of 6-drink measure for both women and men was also used[18]. In the present study, we adopted the definition using ≥ 4/5 drinks for women/wen and found that the 9.2% overall prevalence of binge drink in Zhejiang was higher than in Hong Kong (7.1%)[15]. The higher prevalence in boys than in girls was consistent with results from other studies[15 18 34], but different from study in Korea, where no sex difference observed[14]. The vocational high school had the higher prevalence of binge drinking than the other types of schools. A possible explanation was that as compared to middle school and academic high school students, vocational high school students are going to step into society after graduation, and so they maybe need more social communication. Meanwhile, drinking alcohol was considered as an important skill of social communication[17]. In subgroups analysis, we found the top three subgroups with the highest prevalence of binge drinking was current drinker subgroup (51.2%), students with sexual experience (39.2%) subgroup, students committing suicide subgroup (31.5%). This suggests that students with those behaviour should be identified as target population for at-risk probability of binge drinking.

Association of demographic factors of binge drinking

No association was found between parental educational levels with binge drinking in our study. In a systematic review including 20 studies from 10 countries or areas, parental socioeconomic status (SES), defined as the educational level, income, or occupation, was weakly but positively associated with binge drinking in developing countries. However, such association was not found in developed countries[35]. Previous study reported that non-intact family structure was associated with alcohol drinking due to low family attachment or insufficient parent-child communication[24 25]. Contrary to the expectations, no such association was found in our study. This may be related to somewhat different parental alcohol-specific rules. Van den et al. found that adolescents were less likely to drink under strict alcohol-specific rules at homes despite non-intact families[36].

Association of behavioral factors of binge drinking

In a cohort study, 89 university students were followed up for two years, and found persistent binge drinking was associated with verbal memory and monitoring diffculties[37]. This might be a possible reason for the positive association we observed between poor academic performance and binge drinking (i.e. binge drinking causes poor academic performance). Several earlier studies also documented that poorer performance among binge drinking students on neuropsychological tasks assessing inhibitory control, cognitive interference, sustained attention, verbal working memory and episodic declarative memory[38-41], functions known to be supported

For peer review only - http://bmjopen.bmj.com/site/about/guidelines.xhtml

by prefrontal and/or hippocampal regions which might be possible biological mechanism underlying the association between poor performance and binge drinking.

Buscemi et al. found that there were a positive relationship between moderate physical activity and alcohol use for males, but not for females[42]. Another study documented that positive relationship between vigorous physical activity and alcohol use was stronger for those who were younger than those who were older[43]. Our study showed that boys being physically active were 1.3 times more likely to binge drink in comparison with boys being not physically active. It is unclear whether physical activity leads to increase odds of binge drink or the other way around (i.e. binge drinking causes increased physical activity). One possible hypothesis might be that boys being physically active are inclined to choose to binge drink as a way of relaxation. Further prospective studies are warranted to ascertain the causal association between physical activity and binge drinking and underlying mechanism.

In our study, nearly one out of five students spent more than 4 hours per day screen time, implying the more and more serious problem in China. Previous studies mainly focused on the relation between television watching with diseases or other factors[44-47]. However, with the exponential growth of electronic screen products, such measures of screen time might underestimate screen time[48]. To our best knowledge, this is the first study to examine association between adolescent binge drinking and screen time, and found that excessive exposure to electronic screen products was positively associated with binge drinking.

Alcohol drinking can damage neurons, decrease neurogenesis, and cause cognitive and affective dysfunction, especially in adolescents[49]. Laboratory evidence proved that decreased neurogenesis resulted in depression-like behaviours in rats[50 51]. The relationship of alcohol use with mental health was bi-directional. Rohde et al. reported that the onset of psychiatric disorder preceded the onset of alcoholism[52], while Berglund et al. reported that depression follow alcoholism[53]. However, binge drinking has been shown to be ineffective in improving one's mental health[54], previous study indicated that substance use might be a means of self-medication and of alleviating the negative feelings that emanate from being lonely[55]. In our study, loneliness was positively associated with adolescent binge drinking, but this association was found only among girls, consistent with findings among Arkhangelsk adolescents[56]. Huang et al. reported alcohol drinking was significantly associated with emotional symptoms in girls only[57]. Our findings suggest that preventive strategies against binge drinking for female adolescents should include mental health consultation.

As demonstrated in our study, with the increase of insomnia, the odds of adolescent binge drinking increased. A previous study including 14 089 participants published by Popovici et al. revealed that binge drinking was positively associated with sleep problems (having trouble falling asleep or staying asleep), independent of psychiatric conditions. There was a dose-response

For peer review only - http://bmjopen.bmj.com/site/about/guidelines.xhtml

relationship between sleep problems and frequency of binge drinking[58], which was in accordance with our study. Rishi et al. found that binge drinking could reverse sleep-wake cycle in rats and produce insomnia symptom[59], which might be possible mechanism between insomnia and binge drinking of students.

Alcohol drinking is a well-established risk factor of suicidal attempts[60 61]. Acute intoxication may be a greater risk factor for suicide than chronic alcohol use[60], suggesting that binge drinking may produces rapidly acute intoxication and elevate the risk of suicide. In our study, suicide attempt was positively correlated with binge drinking. Consistent with previous studies, binge drinking was related to the use of cigarettes[62 63]. In our study, current smoking had a strongest effect on the odds of binge drinking, with boys and girls smokers having over 5 and 6 times higher possibility to binge drink. Adolescents who drink alcohol were more likely to be involved in interpersonal conflicts and violence[23]. Our result revealed that both fighting and being bullied were positively related with adolescent binge drinking, which highlights the need for adolescent violence prevention programs that focus on the reduction of alcohol abuse. We found positive association between binge drinking and sexual experiences, which was in correspondence with earlier study[23]. Lewis et al. reported that alcohol facilitated formation of intimate relationship[64]. In addition, previous study reported that binge drinking of adolescents was also associated with higher rates of unwanted pregnancy, sexually transmitted infections, and infertility[8].

Limitations

Our study had several limitations. First, the study was based on a cross-sectional study, which did not allow us establish the casual relationship between factors and binge drinking. Second, all of the data was self-reported by students, alcohol consumption might not be accurately reported because of recall and social desirability biases. Third, only students attending schools participated in the survey. Given the possibility that students being expelled, suspended, or dropped out from school were more likely to binge drink, the overall prevalence observed in this study may represent a conservative estimate of the real situation in the study area.

Conclusions

Despite these limitations, our study identified the correlates of binge drinking among middle and high school students in Zhejiang and quantified their strength of associations, and provided information for preventive strategies against binge drinking. Efforts to prevent binge drinking may need to address a cluster of correlating factors, including cigarettes smoking, excessive exposure to electronic screen products, suicide attempt, fighting, being bullied, loneliness, insomnia, sexual behaviour, and so on. The findings would be instructive for healthcare providers to detect students with high-risk of binge drinking, which will aid in planning intervention measures for at-risk students.

For peer review only

Acknowledgements The authors would like to thank all the students, parents, teachers and local officials for their participation, assistance and co-operation.

Contributor HW designed the study, and collected and analyzed the data with MY. JZ and RH were involved in data interpretation. HD took part in data analysis and revised the manuscript. MW was involved in data collection. All the authors have read and approved the final submitted version.

Competing interests None declared.

Funding The work was supported by grant (2016YFC0900502) from National Key Research and Development Program of China.

Ethics approval Ethics Committee of Zhejiang Provincial Centre for Disease Control and Prevention.

Data sharing statement No additional data are available

For peer review only

References

1. Rehm J, Mathers C, Popova S, et al. Global burden of disease and injury and economic cost attributable to alcohol use and alcohol-use disorders. *Lancet* 2009;373(9682):2223-33. doi: 10.1016/S0140-6736(09)60746-7
2. Collaborators GBDRF. Global, regional, and national comparative risk assessment of 84 behavioural, environmental and occupational, and metabolic risks or clusters of risks, 1990-2016: a systematic analysis for the Global Burden of Disease Study 2016. *Lancet* 2017;390(10100):1345-422. doi: 10.1016/S0140-6736(17)32366-8
3. Bingham CR, Shope JT, Parow JE, et al. Crash types: markers of increased risk of alcohol-involved crashes among teen drivers. *J Stud Alcohol Drugs* 2009;70(4):528-35.
4. Scott KD, Schafer J, Greenfield TK. The role of alcohol in physical assault perpetration and victimization. *J Stud Alcohol* 1999;60(4):528-36.
5. Foran HM, O'Leary KD. Alcohol and intimate partner violence: a meta-analytic review. *Clin Psychol Rev* 2008;28(7):1222-34. doi: 10.1016/j.cpr.2008.05.001
6. Katon W, Richardson L, Russo J, et al. Depressive symptoms in adolescence: the association with multiple health risk behaviors. *Gen Hosp Psychiatry* 2010;32(3):233-9. doi: 10.1016/j.genhosppsych.2010.01.008
7. Marmorstein NR. Longitudinal associations between alcohol problems and depressive symptoms: early adolescence through early adulthood. *Alcohol Clin Exp Res* 2009;33(1):49-59. doi: 10.1111/j.1530-0277.2008.00810.x
8. Stolle M, Sack PM, Thomasius R. Binge drinking in childhood and adolescence: epidemiology, consequences, and interventions. *Dtsch Arztebl Int* 2009;106(19):323-8. doi: 10.3238/arztebl.2009.0323
9. Degenhardt L, O'Loughlin C, Swift W, et al. The persistence of adolescent binge drinking into adulthood: findings from a 15-year prospective cohort study. *BMJ Open* 2013;3(8):e003015. doi: 10.1136/bmjopen-2013-003015

For peer review only - http://bmjopen.bmj.com/site/about/guidelines.xhtml

10. Viner RM, Taylor B. Adult outcomes of binge drinking in adolescence: findings from a UK national birth cohort. *J Epidemiol Community Health* 2007;61(10):902-7. doi: 10.1136/jech.2005.038117
11. Kim JH, Lee S, Chow J, et al. Prevalence and the factors associated with binge drinking, alcohol abuse, and alcohol dependence: a population-based study of Chinese adults in Hong Kong. *Alcohol Alcohol* 2008;43(3):360-70. doi: 10.1093/alcalc/agm181
12. The ESPAD Group: ESPAD Report 2015 Result from the European School Survey Project on Alcohol and Other Drugs. Lisbon, 2016.
13. Esser MB, Clayton H, Demissie Z, et al. Current and Binge Drinking Among High School Students - United States, 1991-2015. *MMWR Morb Mortal Wkly Rep* 2017;66(18):474-78. doi: 10.15585/mmwr.mm6618a4
14. Chung SS, Joung KH. Risk factors of heavy episodic drinking among Korean adolescents. *J Psychiatr Ment Health Nurs* 2013;20(8):665-71. doi: 10.1111/j.1365-2850.2012.01954.x
15. Huang R, Ho SY, Wang MP, et al. Sociodemographic risk factors of alcohol drinking in Hong Kong adolescents. *J Epidemiol Community Health* 2016;70(4):374-9. doi: 10.1136/jech-2015-206418
16. Qian L, Yi HG, Tian BC, et al. [Alcohol consumption and drug use among middle school students aged 13-15 in 4 cities of China]. *Zhonghua Liu Xing Bing Xue Za Zhi* 2006;27(6):483-7.
17. Xing Y, Ji C, Zhang L. Relationship of binge drinking and other health-compromising behaviors among urban adolescents in China. *J Adolesc Health* 2006;39(4):495-500. doi: 10.1016/j.jadohealth.2006.03.014
18. Lu S, Du S, Hu X, et al. Drinking patterns and the association between socio-demographic factors and adolescents' alcohol use in three metropolises in China. *Int J Environ Res Public Health* 2015;12(2):2037-53. doi: 10.3390/ijerph120202037
19. Au WM, Ho SY, Wang MP, et al. Correlates of pro-drinking practices in drinking parents of adolescents in Hong Kong. *PLoS One* 2015;10(3):e0119554. doi: 10.1371/journal.pone.0119554
20. Wang S, Newman IM, Shell DF. Cultural Orientation and Its Associations with Alcohol Use by University Students in China. *PLoS One* 2016;11(11):e0165858. doi: 10.1371/journal.pone.0165858
21. Newman J, Xue J, Fang X. Alcohol Use and Its Risk Factors Among High School Students in Beijing *Chin J Sch Health* 2004;25(4):385-86.
22. Zuo J, Wang H, Zhou N. Investigation on the the Drinking State and the Influencing Factors in Middle School Students in Wuhan City. *Chin Med Soc* 2001;14(3):10-12.
23. Miller JW, Naimi TS, Brewer RD, et al. Binge drinking and associated health risk behaviors among high school students. *Pediatrics* 2007;119(1):76-85. doi: 10.1542/peds.2006-1517
24. Sokol-Katz J, Dunham R, Zimmerman R. Family structure versus parental attachment in controlling adolescent deviant behavior: a social control model. *Adolescence* 1997;32(125):199-215.
25. Mak KK, Ho SY, Thomas GN, et al. Family structure, parent-child conversation time and substance use among Chinese adolescents. *BMC Public Health* 2010;10:503. doi: 10.1186/1471-2458-10-503
26. Centers for Disease Control and Prevention . 2017 State and Local Youth Risk Behavior Survey; 2017 [Available from: https://www.cdc.gov/healthyyouth/data/yrbs/questionnaires.htm accessed 21/11 2017.
27. World Health Organization.Global school-based students health survey(GSHS) 2015 [updated 7/6. Available from: http://www.who.int/ncds/surveillance/gshs/nepal/en/ accessed 21/11 2017.
28. Newsletter N. NIAAA Council Approves Definition of Binge Drinking. 2004.
29. Pengpid S, Peltzer K. Alcohol use and associated factors among adolescent students in Thailand. *West Indian Med J* 2012;61(9):890-6.

For peer review only

For peer review only - http://bmjopen.bmj.com/site/about/guidelines.xhtml

30. Wechsler H, Austin SB. Binge drinking: the five/four measure. *J Stud Alcohol* 1998;59(1):122-4.
31. Wechsler H, Dowdall GW, Davenport A, et al. A gender-specific measure of binge drinking among college students. *Am J Public Health* 1995;85(7):982-5.
32. Wechsler H, Nelson TF. Binge drinking and the American college student: what's five drinks? *Psychol Addict Behav* 2001;15(4):287-91.
33. Baraona E, Abittan CS, Dohmen K, et al. Gender differences in pharmacokinetics of alcohol. *Alcohol Clin Exp Res* 2001;25(4):502-7.
34. Tavolacci MP, Boerg E, Richard L, et al. Prevalence of binge drinking and associated behaviours among 3286 college students in France. *BMC Public Health* 2016;16:178. doi: 10.1186/s12889-016-2863-x
35. Kwok KH, Yuan SN. Parental socioeconomic status and binge drinking in adolescents: A systematic review. *Am J Addict* 2016 doi: 10.1111/ajad.12461
36. van den Eijnden R, Mheen D, Vet R, et al. Alcohol-specific parenting and adolescents' alcohol-related problems: the interacting role of alcohol availability at home and parental rules. *J Stud Alcohol Drugs* 2011;72(3):408-17.
37. Mota N, Parada M, Crego A, et al. Binge drinking trajectory and neuropsychological functioning among university students: a longitudinal study. *Drug Alcohol Depend* 2013;133(1):108-14. doi: 10.1016/j.drugalcdep.2013.05.024
38. Heffernan T, Clark R, Bartholomew J, et al. Does binge drinking in teenagers affect their everyday prospective memory? *Drug Alcohol Depend* 2010;109(1-3):73-8. doi: 10.1016/j.drugalcdep.2009.12.013
39. Ehlers CL, Phillips E, Finnerman G, et al. P3 components and adolescent binge drinking in Southwest California Indians. *Neurotoxicol Teratol* 2007;29(1):153-63. doi: 10.1016/j.ntt.2006.11.013
40. Garcia-Moreno LM, Exposito J, Sanhueza C, et al. [Prefrontal activity and weekend alcoholism in the young]. *Adicciones* 2008;20(3):271-9.
41. Johnson CA, Xiao L, Palmer P, et al. Affective decision-making deficits, linked to a dysfunctional ventromedial prefrontal cortex, revealed in 10th grade Chinese adolescent binge drinkers. *Neuropsychologia* 2008;46(2):714-26. doi: 10.1016/j.neuropsychologia.2007.09.012
42. Buscemi J, Martens MP, Murphy JG, et al. Moderators of the relationship between physical activity and alcohol consumption in college students. *J Am Coll Health* 2011;59(6):503-9. doi: 10.1080/07448481.2010.518326
43. Lisha NE, Martens M, Leventhal AM. Age and gender as moderators of the relationship between physical activity and alcohol use. *Addict Behav* 2011;36(9):933-6. doi: 10.1016/j.addbeh.2011.04.003
44. Alghadir AH, Gabr SA, Iqbal ZA. Television watching, diet and body mass index of school children in Saudi Arabia. *Pediatr Int* 2016;58(4):290-4. doi: 10.1111/ped.12834
45. Al-Ghamdi SH. The association between watching television and obesity in children of school-age in Saudi Arabia. *J Family Community Med* 2013;20(2):83-9. doi: 10.4103/2230-8229.114767
46. Mamun AA, O'Callaghan MJ, Williams G, et al. Television watching from adolescence to adulthood and its association with BMI, waist circumference, waist-to-hip ratio and obesity: a longitudinal study. *Public Health Nutr* 2013;16(1):54-64. doi: 10.1017/S1368980012002832
47. Mekary RA, Lucas M, Pan A, et al. Isotemporal substitution analysis for physical activity, television watching, and risk of depression. *Am J Epidemiol* 2013;178(3):474-83. doi: 10.1093/aje/kws590
48. Jiang XX, Hardy LL, Ding D, et al. Recreational screen-time among Chinese adolescents: a cross-sectional study. *J Epidemiol* 2014;24(5):397-403.
49. Guerri C, Pascual M. Mechanisms involved in the neurotoxic, cognitive, and neurobehavioral effects of alcohol consumption during adolescence. *Alcohol* 2010;44(1):15-26. doi: 10.1016/j.alcohol.2009.10.003

For peer review only - http://bmjopen.bmj.com/site/about/guidelines.xhtml

50. Santarelli L, Saxe M, Gross C, et al. Requirement of hippocampal neurogenesis for the behavioral effects of antidepressants. *Science* 2003;301(5634):805-9. doi: 10.1126/science.1083328
51. Malberg JE, Eisch AJ, Nestler EJ, et al. Chronic antidepressant treatment increases neurogenesis in adult rat hippocampus. *J Neurosci* 2000;20(24):9104-10.
52. Rohde P, Lewinsohn PM, Seeley JR. Psychiatric comorbidity with problematic alcohol use in high school students. *J Am Acad Child Adolesc Psychiatry* 1996;35(1):101-9. doi: 10.1097/00004583-199601000-00018
53. Berglund M, Ojehagen A. The influence of alcohol drinking and alcohol use disorders on psychiatric disorders and suicidal behavior. *Alcohol Clin Exp Res* 1998;22(7 Suppl):333S-45S.
54. Kim JH, Chan KW, Chow JK, et al. University binge drinking patterns and changes in patterns of alcohol consumption among chinese undergraduates in a Hong Kong university. *J Am Coll Health* 2009;58(3):255-65. doi: 10.1080/07448480903295318
55. Page RM, Dennis M, Lindsay GB, et al. Psychosocial distress and substance use among adolescents in four countries: Philippines, China, Chile, and Namibia. *Youth & Society* 2011;43(3):900-30.
56. Stickley A, Koyanagi A, Koposov R, et al. Loneliness and health risk behaviours among Russian and U.S. adolescents: a cross-sectional study. *BMC Public Health* 2014;14:366. doi: 10.1186/1471-2458-14-366
57. Huang R, Ho SY, Wang MP, et al. Reported alcohol drinking and mental health problems in Hong Kong Chinese adolescents. *Drug Alcohol Depend* 2016;164:47-54. doi: 10.1016/j.drugalcdep.2016.04.028
58. Popovici I, French MT. Binge drinking and sleep problems among young adults. *Drug Alcohol Depend* 2013;132(1-2):207-15. doi: 10.1016/j.drugalcdep.2013.02.001
59. Sharma R, Bradshaw K, Sahota P, et al. Acute binge alcohol administration reverses sleep-wake cycle in Sprague Dawley rats. *Alcohol Clin Exp Res* 2014;38(7):1941-6. doi: 10.1111/acer.12463
60. Cherpitel CJ, Borges GL, Wilcox HC. Acute alcohol use and suicidal behavior: a review of the literature. *Alcohol Clin Exp Res* 2004;28(5 Suppl):18S-28S.
61. Kaplan MS, Giesbrecht N, Caetano R, et al. Acute alcohol consumption as a contributing factor to suicidal behavior. *Am J Public Health* 2013;103(9):e2-3. doi: 10.2105/AJPH.2013.301422
62. Hale D, Viner R. Trends in the prevalence of multiple substance use in adolescents in England, 1998-2009. *J Public Health (Oxf)* 2013;35(3):367-74. doi: 10.1093/pubmed/fdt022
63. Stickley A, Koyanagi A, Koposov R, et al. Binge drinking among adolescents in Russia: prevalence, risk and protective factors. *Addict Behav* 2013;38(4):1988-95. doi: 10.1016/j.addbeh.2012.12.009
64. Lewis MA, Granato H, Blayney JA, et al. Predictors of hooking up sexual behaviors and emotional reactions among U.S. college students. *Arch Sex Behav* 2012;41(5):1219-29. doi: 10.1007/s10508-011-9817-2

For peer review only

For peer review only - http://bmjopen.bmj.com/site/about/guidelines.xhtml

Table 1 questions comprising variables included in the survey

Variables	Questions and options
Parental education level	What is the highest level of education your father/mother has obtained? (Answer options: primary school or below, middle school, high school, college or university, master graduates or above, unknown)
Parental marital status	What is your parents' current marital status? (Answer options: married, divorced, widowed, separated)
Siblings	Are you the only son/daughter of your parent?(Answer options: yes, no)
Physical activity	During the past 7 days, on how many days were you physically active for a total of at least 60 minutes per day?(Answer options: none, 1 days, 2 days, 3 days, 4 days, 5 days, 6 days, 7 days)
Academic performance	How would you describe your grades in your class?(Answer options: excellent, middle, poor)
Screen time	On an average school day, how many hours do you play video or computer games or use a computer for something that is not school work? (Answer options: none, < 1 h/d, 1 h/d, 2 h/d, 3 h/d, 4 h/d, ≥5 h/d)
Loneliness	During the past 12 months, did you ever feel lonely? (Answer options: never, occasional, sometimes, often, always)
Insomnia	During the past 12 months, have you ever felt worried about something that you cannot fall asleep? (Answer options: never, occasional, sometimes, often, always)
Suicidal attempt	During the past 12 months, how many times did you actually attempt suicide? (Answer options: none, 1 time, 2-3times, 4-5 times, 6 or more times)
Current smoking	During the past 30 days, on how many days did you smoke cigarettes? (Answer options: none, 1-2 days, 3-5 days, 6-9 days, 10-19 days, 20-29 days and all 30 days)
Fighting	During the past 12 months, how many times were you in a physical fight？(Answer options: none, 1 time, 2-3times, 4-5 times, 6-7 times, 8-9 times, 10-11 times, 12 or more times)
Being bullied	During the past 12 months, how many times has someone threatened or injured you with a weapon such as a gun, knife, or club on school property? (Answer options: none, 1 time, 2-3times, 4-5 times, 6-7 times, 8-9 times, 10-11 times, 12 or more times)
Sexual experience	Have you ever had sexual intercourse? (Answer options: yes, no)

For peer review only - http://bmjopen.bmj.com/site/about/guidelines.xhtml

Table 2 characteristics of adolescents from Zhejiang (N=23543)

Characteristics	Total	Boys	Girls
Age range (years)			
≤13	5 159 (20.9)	2 689 (21.1)	2 470 (20.8)
14	4 300 (17.8)	2 192 (17.7)	2 108 (17.8)
15	3 730 (16.5)	1 905 (16.5)	1 825 (16.6)
≥16	10 354 (44.8)	5 282 (44.7)	5 072 (44.8)
Area			
Urban	9 022 (31.8)	4 544 (31.8)	4 478 (31.9)
Rural	14 521 (68.2)	7 524 (68.2)	6 997 (68.1)
Types of school			
Middle school	12 207 (51.8)	6 364 (52.4)	5 843 (51.0)
Academic high school	6 477 (26.1)	3 223 (25.1)	3 254 (27.3)
Vocational high school	4 859 (22.1)	2 481 (22.5)	2 378 (21.7)
Parental marital status			
Married	21 151 (90.1)	10 924 (90.9)	10 227 (89.2)
Others	2 392 (9.9)	1 144 (9.1)	1 248 (10.8)
Paternal education level			
Primary or below	13 568 (60.0)	6 908 (59.5)	6 660 (60.5)
Middle or high school	5 100 (20.8)	2 628 (20.8)	2 472 (20.7)
College or above	3 129 (11.4)	1 575 (11.2)	1 554 (11.7)
Unknown	1 746 (7.8)	957 (8.5)	789 (7.1)
Maternal education level			
Primary or below	14 530 (63.9)	7 292 (62.5)	7 238 (65.5)
Middle or high school	4 363 (17.7)	2 271 (18.0)	2 092 (17.3)
College or above	2 736 (10.0)	1 392 (9.8)	1 344 (10.2)
Unknown	1 914 (8.4)	1 113 (9.7)	801 (7.0)
Having siblings			
Yes	12 137 (54.8)	5 263 (46.6)	6 874 (63.8)
No	11 406 (45.2)	6 805 (53.4)	4 601 (36.2)
Academic performance			
Excellent	5 448 (22.5)	2 731 (21.7)	2 717 (23.3)
Middle	11 765 (50.5)	5 727 (48.1)	6 038 (53.1)
Bad	6 330 (27.0)	3 610 (30.2)	2 720 (23.6)
Physical activity (d/wk)			
0	4 883 (21.5)	2 079 (18.1)	2 804 (25.2)
1-2	5 690 (24.8)	2 703 (22.9)	2 987 (26.8)
3-5	8 050 (33.0)	4 237 (34.5)	3 813 (31.5)
6-7	4 920 (20.7)	3 049 (24.5)	1 871 (16.5)
Screen time (h/d)			
0	7 255 (31.2)	3 604 (29.7)	3 651 (32.8)
<1	4 009 (15.9)	1 968 (15.1)	2 041 (16.7)
1-4	7 621 (31.4)	3 853 (31.1)	3 768 (31.6)
⩾4	4 658 (21.5)	2 643 (24.1)	2 015 (18.9)
Loneliness			
Never/occasional	15 122 (64.1)	8 082 (66.8)	7 040 (61.2)
Sometimes	5 783 (24.7)	2 698 (22.6)	3 085 (26.8)
Often/always	2 638 (11.2)	1 288 (10.6)	1 350 (12.0)
Insomnia			
Never/occasional	18 682 (78.6)	9 880 (81.2)	8 802 (75.8)
Sometimes	3 765 (16.7)	1 653 (14.3)	2 112 (19.3)
Often/always	1 096 (4.7)	535 (4.5)	561 (4.9)
Suicide attempt			
Yes	854 (3.6)	337 (2.9)	517 (4.4)
No	22 689 (96.4)	1 1731 (97.1)	10 958 (95.6)
Current smoking			
Yes	1 247 (5.5)	968 (8.2)	279 (2.5)
No	22 296 (94.5)	1 1100 (91.8)	11 196 (97.5)
Fighting			
Yes	3 707 (15.6)	2 966(24.1)	741 (6.4)
No	19 836 (84.4)	9 102(75.9)	10 734 (93.6)
Being bullied			
Yes	3 041 (13.2)	1 872(15.7)	1169 (10.5)
No	20 502 (86.8)	10 196(84.3)	10 306 (89.5)
Sexual experience			
Yes	861 (3.9)	591 (5.1)	270(2.5)
No	22 682 (96.1)	11 477 (94.9)	11 205(97.5)

Number in brackets were weighted proportion

For peer review only - http://bmjopen.bmj.com/site/about/guidelines.xhtml

Table 3 weighted prevalence of current drinking and binge drinking by different subgroups

Characteristics	Current drinking			Binge drinking		
	Prevalence (%)*	Chi-Square	*P*	Prevalence (%)*	Chi-Square	*P*
Sex		93.65†	<.0001		80.46†	<.0001
Boys	27.0 (25.6-28.5)			11.8 (10.8-12.9)		
Girls	18.2 (16.8-19.6)			6.5 (5.6-7.3)		
Area		2.38†	0.12		0.62†	0.4314
Urban	24.4 (21.9-26.9)			9.8 (8.2-11.4)		
Rural	22.0 (20.5-23.5)			9.0 (8.0-10.0)		
Types of school		319.93†	<.0001		278.29†	<.0001
Middle school	17.5 (16.2-18.8)			6.3 (5.6-7.0)		
Academic high school	22.1 (20.4-23.8)			7.7 (6.8-8.7)		
Vocational high school	35.9 (33.7-38.0)			17.9 (15.9-20.0)		
Academic performance		68.9‡	<.0001		51.7‡	<.0001
Excellent	18.2 (16.4-20.0)			6.9 (5.7-8.1)		
Middle	21.6 (20.2-22.9)			8.5 (7.6-9.3)		
Poor	28.8 (26.9-30.6)			12.7 (11.5-13.8)		
Physical activity (d/wk)		1.10‡	0.2977		2.0‡	0.1555
0	23.2 (21.6-24.8)			9.7 (8.7-10.8)		
1-2	22.0 (20.5-23.5)			8.5 (7.4-9.5)		
3-5	21.7 (20.1-23.2)			8.3 (7.3-9.3)		
6-7	25.0 (23.0-26.9)			11.2 (9.9-12.5)		
Screen time (h/d)		330.9‡	<.0001		259.6‡	<.0001
0	16.1 (14.8-17.3)			5.5 (4.8-6.3)		
<1	16.8 (15.3-18.3)			5.5 (4.7-6.4)		
1-4	23.3 (21.8-24.7)			8.9 (7.8-9.9)		
≥4	36.1 (34.0-38.1)			17.9 (16.3-19.6)		
Loneliness		149.6‡	<.0001		113.9‡	<.0001
Never/occasional	19.6 (18.4-20.7)			7.5 (6.8-8.2)		
Sometimes	26.2 (24.4-27.9)			10.4 (9.4-11.5)		
Often/always	33.5 (31.4-35.6)			16.5 (14.7-18.3)		
Insomnia		177.2‡	<.0001		205.3‡	<.0001
Never/occasional	20.4 (19.3-21.5)			7.7 (7.0-8.3)		
Sometimes	29.3 (27.3-31.2)			13.0 (11.6-14.5)		
Often/always	39.2 (35.8-42.5)			22.7 (19.9-25.5)		
Suicide attempt		204.61†	<.0001		288.99†	<.0001
Yes	48.4 (44.2-52.6)			31.5 (27.4-35.6)		
No	21.8 (20.6-23.0)			8.4 (7.7-9.2)		
Current smoking		7335.31†	<.0001		3720.33†	<.0001
Yes	73.3 (70.5-76.0)			51.2 (47.2-55.2)		
No	19.8 (18.9-20.8)			6.8 (6.3-7.4)		
Fighting		257.16†	<.0001		307.89†	<.0001
Yes	40.3 (37.2-43.4)			21.5 (19.1-23.9)		
No	19.5 (18.5-20.6)			7.0 (6.3-7.6)		
Being bullied		49.13†	<.0001		38.81†	<.0001
Yes	31.1 (28.1-34.1)			14.1 (12.0-16.2)		
No	21.5 (20.4-22.6)			8.5 (7.8-9.2)		
Sexual experience		526.80†	<.0001		500.40†	<.0001
Yes	59.1 (54.5-63.6)			39.2 (33.8-44.5)		
No	21.3 (20.2-22.4)			8.1 (7.4-8.7)		

*Based on the weighted data. †: Rao-Scott Chi-Square; ‡: Trend for Chi-Square

For peer review only - http://bmjopen.bmj.com/site/about/guidelines.xhtml

Table 4 crude and adjusted odd ratio of factors associated with binge drinking among adolescents in China

Variable	Boys (N=12 068)		Girls (N=11 475)	
	COR(95%CI)	AOR(95%CI)	COR(95%CI)	AOR(95%CI)
Age groups (ref: ≤13 years)				
14	**1.95 (1.52-2.50) &**	**1.95 (1.51-2.53) &**	1.12 (0.77-1.65)	1.16 (0.76-1.77)
15	**2.70 (2.06-3.53) &**	**2.21(1.67-2.90) &**	**1.46 (1.01-2.12) #**	1.12 (0.74-1.69)
≥16	**3.89 (3.07-4.92) &**	**2.10(1.55-2.84) &**	**1.68 (1.13-2.50) ***	1.03 (0.60-1.77)
Rural (ref: Urban)	1.00 (0.77-1.29)		0.77 (0.57-1.04)	
Types of school (ref: Middle school)				
Academic high school	**1.62 (1.33-1.96) &**	**1.48(1.15-1.90) ***	0.78 (0.60-1.00)	0.99 (0.64-1.55)
Vocational high school	**3.65 (2.91-4.58) &**	**2.09(1.59-2.74) &**	**2.71 (2.07-3.55) &**	**2.18 (1.49-3.19) $**
Parental marital status (ref: Married)				
Others	**1.32 (1.08-1.61) ***	0.91(0.73-1.13)	**2.07 (1.67-2.57) &**	1.25 (0.95-1.65)
Paternal education level				
(ref: Primary or below)				
Middle or high school	1.00 (0.84-1.20)	1.13(0.93-1.38)	0.84 (0.68-1.05)	1.01 (0.80-1.27)
College or above	**0.76 (0.60-0.95) #**	0.94(0.75-1.18)	**0.66 (0.49-0.90) ***	0.86 (0.62-1.19)
Unknown	0.91 (0.72-1.16)	0.99(0.78-1.26)	1.23 (0.89-1.71)	1.25 (0.88-1.79)
Maternal education level				
(ref: Primary or below)				
Middle or high school	0.99 (0.84-1.17)		0.99 (0.77-1.27)	
College or above	0.78 (0.61-1.00)		0.77 (0.56-1.05)	
Unknown	0.82 (0.64-1.06)		1.11 (0.77-1.61)	
Siblings (ref: No)	0.92 (0.80-1.06)		0.99 (0.80-1.22)	
Academic performance (ref: Excellent)				
Middle	**1.23 (1.01-1.50) #**	1.13 (0.93-1.36)	1.28 (0.95-1.73)	1.17 (0.87-1.58)
Bad	**1.82 (1.49-2.22) &**	**1.27 (1.03-1.56) #**	**1.97 (1.43-2.70)** &	1.30 (0.94-1.79)
Physical activity (ref: 0 d/wk)				
1-2 d/w	0.82 (0.66-1.02)	0.90 (0.70-1.16)	0.86 (0.70-1.06)	
3-5 d/w	**0.79 (0.65-0.96) #**	0.93 (0.75-1.14)	0.78 (0.60-1.02)	
6-7 d/w	1.09 (0.90-1.33)	**1.33 (1.07-1.66) #**	0.95 (0.74-1.21)	
Screen time (ref: 0 h/d)				
<1 h/d	0.90 (0.72-1.12)	0.94 (0.75-1.17)	1.27 (0.88-1.84)	1.18 (0.81-1.73)
1-4 h/d	**1.47 (1.19-1.82) $**	1.14 (0.91-1.43)	**2.13 (1.62-2.80) &**	**1.61 (1.20-2.17) ***

For peer review only

≥ 4 h/d	**2.79 (2.28-3.41) &**	**1.44 (1.16-1.79) $**	**5.91 (4.53-7.71) &**	**2.59 (1.93-3.48) &**
Loneliness (ref: Never/Occasionally)				
Sometimes	**1.42 (1.23-1.63) &**	1.08 (0.91-1.28)	**1.69 (1.37-2.08) &**	1.23 (0.96-1.57)
Often/Always	**2.25 (1.87-2.72) &**	1.18 (0.90-1.56)	**3.14 (2.51-3.93) &**	**1.43 (1.05-1.95) #**
Insomnia (ref: Never/Occasionally)				
Sometimes	**1.97 (1.68-2.32) &**	**1.41 (1.13-1.77) ***	**1.92 (1.59-2.31) &**	**1.33 (1.06-1.65) $**
Often/Always	**2.89 (2.29-3.64) &**	**1.69 (1.23-2.31) ***	**5.18 (4.05-6.62) &**	**2.23 (1.63-3.06) &**
Suicide attempt (ref: No)	**4.14 (3.24-5.30) &**	**2.37 (1.73-3.25) &**	**7.35 (5.51-9.81) &**	**3.36 (2.48-4.56) &**
Current smoking (ref: No)	**11.24 (9.13-13.84) &**	**5.21 (4.13-6.58) &**	**20.27 (15.32-26.83) &**	**6.68 (4.64-9.60) &**
Fighting (ref: No)	**2.78 (2.37-3.27) &**	**2.18 (1.81-2.62) &**	**5.07 (4.04-6.37) &**	**2.48 (1.87-3.29) &**
Being bullied (ref: No)	**1.59 (1.34-1.89) &**	**1.23 (1.03-1.46) #**	**1.81 (1.27-2.59) ***	1.04 (0.69-1.56)
Sexual experience (ref: No)	**6.92 (5.33-8.98) &**	**2.33 (1.69-3.20) &**	**6.60 (4.94-8.83) &**	**1.82 (1.22-2.72) ***

Bold numbers represent significant results

COR: Crude odds ratio. AOR: Adjusted odds ratios. CI: confidence intervals. #: P<0.05. *: P<0.01. $: P<0.001. &: P<0.0001

AOR is adjusted for all other covariates in the model

For peer review only

STROBE Statement—checklist of items that should be included in reports of observational studies

	Item No	Recommendation
**Title and abstract**	1 **YES Title:page1 Abstract: page2**	(*a*) Indicate the study's design with a commonly used term in the title or the abstract
		(*b*) Provide in the abstract an informative and balanced summary of what was done and what was found
**Introduction**		
Background/rationale	2 **YES Page4-5**	Explain the scientific background and rationale for the investigation being reported
Objectives	3 **YES Page 2**	State specific objectives, including any prespecified hypotheses
**Methods**		
Study design	4 **YES Page4-5**	Present key elements of study design early in the paper
Setting	5 **YES Page4-5**	Describe the setting, locations, and relevant dates, including periods of recruitment, exposure, follow-up, and data collection
Participants	6 **YES Page4-5**	(*a*) *Cohort study*—Give the eligibility criteria, and the sources and methods of selection of participants. Describe methods of follow-up<br>*Case-control study*—Give the eligibility criteria, and the sources and methods of case ascertainment and control selection. Give the rationale for the choice of cases and controls<br>*Cross-sectional study*—Give the eligibility criteria, and the sources and methods of selection of participants
		(*b*) *Cohort study*—For matched studies, give matching criteria and number of exposed and unexposed<br>*Case-control study*—For matched studies, give matching criteria and the number of controls per case
Variables	7 **YES Page6-7**	Clearly define all outcomes, exposures, predictors, potential confounders, and effect modifiers. Give diagnostic criteria, if applicable
Data sources/ measurement	8* **YES Page 5，18**	For each variable of interest, give sources of data and details of methods of assessment (measurement). Describe comparability of assessment methods if there is more than one group
Bias	9 **YES Page 7**	Describe any efforts to address potential sources of bias
Study size	10 **YES Page5-6**	Explain how the study size was arrived at
Quantitative variables	11 **YES**	Explain how quantitative variables were handled in the analyses. If applicable, describe which groupings were chosen and why
Statistical methods	12 **YES Page 7**	(*a*) Describe all statistical methods, including those used to control for confounding
		(*b*) Describe any methods used to examine subgroups and interactions
		(*c*) Explain how missing data were addressed
		(*d*) *Cohort study*—If applicable, explain how loss to follow-up was addressed<br>*Case-control study*—If applicable, explain how matching of cases and

For peer review only - http://bmjopen.bmj.com/site/about/guidelines.xhtml

For peer review only

		controls was addressed<br>*Cross-sectional study*—If applicable, describe analytical methods taking account of sampling strategy
		(*e*) Describe any sensitivity analyses
**Results**		
Participants	13*<br>**YES**<br>**Page5**	(a) Report numbers of individuals at each stage of study—eg numbers potentially eligible, examined for eligibility, confirmed eligible, included in the study, completing follow-up, and analysed
		(b) Give reasons for non-participation at each stage
		(c) Consider use of a flow diagram
Descriptive data	14*<br>**YES**<br>**Page 8**	(a) Give characteristics of study participants (eg demographic, clinical, social) and information on exposures and potential confounders
		(b) Indicate number of participants with missing data for each variable of interest
		(c) *Cohort study*—Summarise follow-up time (eg, average and total amount)
Outcome data	15*<br>**YES**<br>**Page 8-9**	*Cohort study*—Report numbers of outcome events or summary measures over time
		Case-control study—Report numbers in each exposure category, or summary measures of exposure
		Cross-sectional study—Report numbers of outcome events or summary measures
Main results	16<br>**YES**<br>**Page8-9**	(*a*) Give unadjusted estimates and, if applicable, confounder-adjusted estimates and their precision (eg, 95% confidence interval). Make clear which confounders were adjusted for and why they were included
		(*b*) Report category boundaries when continuous variables were categorized
		(*c*) If relevant, consider translating estimates of relative risk into absolute risk for a meaningful time period
Other analyses	17<br>**No**	Report other analyses done—eg analyses of subgroups and interactions, and sensitivity analyses
**Discussion**		
Key results	18 **YES Page 9-12**	Summarise key results with reference to study objectives
Limitations	19<br>**YES**<br>**Page 12**	Discuss limitations of the study, taking into account sources of potential bias or imprecision. Discuss both direction and magnitude of any potential bias
Interpretation	20 **YES page 9-12**	Give a cautious overall interpretation of results considering objectives, limitations, multiplicity of analyses, results from similar studies, and other relevant evidence
Generalisability	21 **YES Page12**	Discuss the generalisability (external validity) of the study results
**Other information**		
Funding	22 **YES Page 13**	Give the source of funding and the role of the funders for the present study and, if applicable, for the original study on which the present article is based

*Give information separately for cases and controls in case-control studies and, if applicable, for exposed and unexposed groups in cohort and cross-sectional studies.

For peer review only

For peer review only - http://bmjopen.bmj.com/site/about/guidelines.xhtml

Note: An Explanation and Elaboration article discusses each checklist item and gives methodological background and published examples of transparent reporting. The STROBE checklist is best used in conjunction with this article (freely available on the Web sites of PLoS Medicine at http://www.plosmedicine.org/, Annals of Internal Medicine at http://www.annals.org/, and Epidemiology at http://www.epidem.com/). Information on the STROBE Initiative is available at www.strobe-statement.org.

For peer review only

BMJ Open

Binge Drinking and Associated Factors among School Students: A Cross-sectional Study in Zhejiang Province, China

Journal:	*BMJ Open*
Manuscript ID	bmjopen-2017-021077.R2
Article Type:	Research
Date Submitted by the Author:	27-Feb-2018
Complete List of Authors:	Wang, Hao; Zhejiang Provincial Center for Diseases Control and Prevention, Department of NCDs Control and Prevention<br>Hu, Ru-Ying; Zhejiang Provincial Center for Diseases Control and Prevention, Department of NCDs Control and Prevention<br>Zhong, Jie-Ming; Zhejiang Provincial Center for Diseases Control and Prevention, Department of NCDs Control and Prevention<br>Du, Huaidong; University of Oxford, Clinical Trial Service Unit & Epidemiological Studies Unit (CTSU), Nuffield Department of Population Health; University of Oxford, Medical Research Council Population Health Research Unit, Nuffield Department of Population Health<br>Bragg, Fiona; University of Oxford, Nuffield Department of Population Health<br>Wang, Meng; Zhejiang Provincal Center for Disease Control and Prevention, Department of NCDs Control and Prevention<br>Yu, Min; Zhejiang Provincial Center for Diseases Control and Prevention
**Primary Subject Heading**:	Public health
Secondary Subject Heading:	Public health
Keywords:	Binge drinking, Alcohol, Adolescents, Factors

SCHOLARONE™
Manuscripts

Binge Drinking and Associated Factors among School Students: A Cross-sectional Study in Zhejiang Province, China

Hao Wang[1], Ruying Hu[1], Jieming Zhong[1], Huaidong Du[2, 3], Bragg Fiona[2], Wang Meng[1], Min Yu[1]

Author affiliations

1. Department of NCDs Control and Prevention, Zhejiang Pcial Center for Diseases Control and Prevention, Hangzhou, China

2. Clinical Trial Service Unit & Epidemiological Studies Unit (CTSU), Nuffield Department of Population Health, University of Oxford, Oxford, UK

3. Medical Research Council Population Health Research Unit, Nuffield Department of Population Health, University of Oxford, Oxford, UK

Correspondence to Min Yu; myu@cdc.zj.cn

For peer review only - http://bmjopen.bmj.com/site/about/guidelines.xhtml

Abstract

Objective: To investigate the prevalence and correlating factors of binge drinking among middle and high school students in Zhejiang Province, China.

Methods: We performed a cross-sectional study using data from a school-based survey. A total of 23 543 (response rate =97.5%) eligible adolescents from 442 different schools (including middle schools, academic high schools and vocational high schools) were asked to fill in an anonymous self-administered behaviour questionnaire between April and May 2017. Multivariable logistic regression models were used to examine the associations of sociodemographic and behavioural factors with binge drinking.

Results: The mean (SD) age of participants was 15.6 (1.7) years and 51.3% were boys. The proportions of students from middle schools, academic high schools and vocational high schools were 51.9%, 27.5% and 20.6%, respectively. Totally, 22.8% (95%CI: 21.6-23.9) of students reported drinking alcohol in the past 30 days and 9.2% (95%CI: 8.5-10.0) of students reported binge drinking (defined as drinking four or more alcoholic drinks in a 1-2 hour period among girls and five or more alcoholic drinks among boys) during the past month. The prevalence of binge drinking was highest among vocational high school students (17.9% vs 6.3% and 7.7% among middle school and academic high school students, respectively). Older age, studying at high school, poor academic performance, higher levels of physical activity, excessive screen-time, loneliness, insomnia, previous suicide attempt, cigarette smoking, fighting, being bullied, and sexual experience were found to be positively associated with adolescent binge drinking.

Conclusions: Binge drinking is common among middle and high school students in Zhejiang, China. Efforts to prevent binge drinking may need to address a cluster of socio-demographic and behavioural factors. Our findings provide information to enable healthcare providers to identify students at high-risk of binge drinking and to inform planning of intervention measures for at-risk students.

Keywords: Binge drinking; Alcohol; Adolescents; Factors

For peer review only - http://bmjopen.bmj.com/site/about/guidelines.xhtml

Strengths and limitations of this study

- This is a school-based study with a relatively large sample size, and a high response rate, and a study population which is representative of Provincial China.
- The study questionnaire includes a wide range of factors, which might help us better understand how to prevent adolescent binge drinking.
- The cross-sectional study design prevents establishment of causal relationships between sociodemographic or behavioural factors and binge drinking.
- Only students attending schools participated in the survey. Students who have been expelled or suspended from school, or who stopped attending, may be more likely to binge drink, and the overall prevalence of binge drinking in our current study might therefore represent an underestimate of the true prevalence.

For peer review only

For peer review only - http://bmjopen.bmj.com/site/about/guidelines.xhtml

Introduction

Alcohol consumption has been identified as an important risk factor for chronic disease and injury [1]. DALYs (disability-adjusted life years) attributable to alcohol consumption have increased by more than 25% between 1990 and 2016, accounting for an estimated 99.2 million worldwide. In 2016, alcohol consumption was responsible for more than 2.8 million deaths worldwide, including approximately 0.7 million in China[2]. Many studies have clearly demonstrated that alcohol misuse among adolescents is associated with motor vehicle collisions[3], violence and crime[4 5], mental health disorders and higher risk of suicide[6-8]. Moreover, alcohol use during early adolescence is a risk factor for later alcohol dependence[9-11].

Binge drinking is defined as episodic excessive drinking. Prevalence of binge drinking among adolescents varies between countries, and is estimated to be 8% in Iceland and 56% in Denmark[12]. Among 25 European countries, prevalence of binge drinking among adolescents rose from 36% in 1995 to 42% in 2007, and then declined to 35% in 2015[12]. In the United States, the prevalence declined from 31.3% in 1991 to 17.7% in 2015[13]. In Asia, the prevalence of heavy episodic drinking (i.e., binge drinking) among grade 7-12 students was 10.3% in Korea[14], whilst 21.2% of students, with mean age 14.7 years, reported drinking alcohol in the past 30 days and 7.1% reported binge drinking in Hong Kong[15]. In mainland China, a study of 7344 middle school students conducted in 4 cities (Beijing, Hangzhou, Wuhan, Urumchi) found that the prevalence of current drinking was 14.4%, and 9.9% of students had experienced drunkenness, with significant regional differences[16]. In another study, conducted in 2004 and including 54 040 students in grades 7-12 from 18 Provincial capitals in China, 25.2% of students reported consuming at least one alcoholic drink and 10.3% reported at least one episode of binge drinking during the 30 days prior to being surveyed[17]. A more recent study, surveying 13 811 high school students from Beijing, Shanghai, and Guangzhou in 2013, found the prevalence of binge drinking was 4.8%[18], although not all school grades were included, likely resulting in underestimation of the true prevalence.

Parental alcohol-related practices have been shown to be important risk factors for adolescent drinking[19]. In traditional Chinese culture, alcohol drinking is both a normal part of the daily diet, especially in rural areas, and an important part of rituals, business occasions, festivals, and special events. Moderate drinking on important occasions is encouraged for adults[20], and children may be given small amounts of alcoholic beverages by parents or grandparents, especially grandfathers[18]. Some previous surveys have found that nearly half of senior high schools students in China have drunk at home with their parents or outside the home with their peers[21 22].

Previous studies have found some inconsistencies in socio-demographic patterns of adolescent binge drinking. For instance, Miller et al. reported that binge drinking prevalence was similar among high school boys and girls in the United States[23], while boys in Hong Kong had higher

For peer review only - http://bmjopen.bmj.com/site/about/guidelines.xhtml

prevalence of binge drinking than girls[15]. Sokol-Katz et al. reported that family structure was not significantly related to behaviours such as alcohol, cigarette, and drug use among middle school students in Florida, after controlling for gender and race/ethnicity[24], while growing up in a non-intact family was associated with substance use among Chinese adolescents in Hong Kong[25].

Zhejiang Province, in the east of China, has a population of 56 million. It has experienced rapid economic development in the past 30 years, which has been associated with increased exposure of adolescents in Zhejiang to western culture. As a result of this, patterns of alcohol use in this area might differ from those in other regions of China. The current study was designed with the aim of examining the prevalence of binge drinking and its correlating factors among students in Zhejiang Province.

Methods

Survey design and participants

The survey utilized a three-stage sampling design. In stage one, 30 counties, including 12 urban areas and 18 rural areas, were sampled randomly from all 90 counties (31 urban and 59 rural) of Zhejiang Province according to socioeconomic status. In stage two, 10 classes of middle school, 5 classes of academic high school, and 5 classes of vocational high school were selected randomly within each chosen counties, respectively. In stage three, all students in the chosen classes were invited to participate in the study. In China, after 6-years of primary school education, children usually attend middle school (i.e., junior high school) for 3-years (grades 7-9). After graduation from middle schools, they enter high school (i.e., senior high school, including academic high schools and vocational high schools) for a further 3-years (grades 10-12). The survey questionnaire was modelled from existing surveys including the Youth Risk Behaviour Survey (YRBS), conducted by the Centers for Disease Control and Prevention (CDC)[26], and the international Global School-based Student Health Survey, supported by the World Health Organization[27]. Survey questions addressed demographic characteristics (including age, gender, parental educational level, parental marital status, number of siblings), tobacco and alcohol use, physical activity, violence, injuries, suicidal behaviours and sexual behaviours. The survey was conducted between April and May 2017. Participants completed the anonymous, self-administrated questionnaire in the classroom.

Sample size calculation

The sample size was calculated by using the formula: $N=deff\times\mu^2\times P\times(1-P)/d^2$. Means and 95% confidence interval (CI; 2-sided for u=1.96) were determined; the prevalence of binge drinking (10%) obtained in the China was used as a measure of probability (p)[17]; the design effect ($deff$) value was set at 3; and the relative error was: $d=r\times1\%$, r=15%. Based on these parameters, the sample size for each stratum was estimated to be 4610 subjects. Because there were 4 strata

For peer review only - http://bmjopen.bmj.com/site/about/guidelines.xhtml

(Areas: urban and rural. Sex: boy and girl), and assuming a potential nonresponse rate of 20%, the final sample size was calculated as 23 050.

Patient and public involvement

Study participants were healthy students and no patients were involved in the study. Students and their parents were not involved in the design and conduct of study. Due to an anonymous survey, our findings will be disseminated to Department of Health and Department of Education, not directly to participating students.

Measures

Outcome variables

For the purposes of this study, alcoholic drinks include beer, wine, wine coolers, rice wine, and liquor such as Chinese liquor, rum, gin, vodka, or whiskey. Current drinking was assessed through the question: "During the past 30 days, on how many days did you have at least one drink of alcohol?". Answer options include: "0 days", "1-2 days", "3-5 days", "6-9 days", "10-19 days", "20-29 days", and "30 days". Participants were considered as current drinkers if they reported drinking alcohol on at least 1-2 days during the past 30 days. Binge drinking was assessed by the question: "During the past 30 days, on how many days did you have 4 or more drinks of alcohol (if you are girl) or 5 or more drinks of alcohol (if you are boy) during a period of 1 to 2 hours?"[28] Answer options include: "0 days", "1 day", "2 days", "3-5 days", "6-9 days", "10-19 days", "≥20 days". Participants were defined as binge drinkers if they answered at least 1 day.

Main covariates

Physical activity was assessed by the question: "During the past 7 days, on how many days were you physically active for a total of at least 60 minutes per day?". Answer options included: "None", "1 day", "2 days", "3 days", "4 days", "5 days", "6 days" and "7 days", and these were further categorized into four groups: "None", "1-2 d/w", "3-5 d/w", and "6-7 d/w". Current smoking was assessed by the question: "During the past 30 days, on how many days did you smoke cigarettes?". Answer options included: "None", "1-2 days", "3-5 days", "6-9 days", "10-19 days", "20-29 days" and "all 30 days". Current smoking was defined as smoking cigarettes on at least 1-2 days in the past 30 days. Screen-time was estimated through the question: "On an average school day, how many hours do you play video or computer games or use a computer for something that is not school work?". Answer options included: "None", "<1 h/d", "1 h/d", "2 h/d", "3 h/d", "4 h/d", "≥5 h/d", and these answers were further categorized into four groups: "None", "<1 h/d", "1-4 h/d", "≥4 h/d". Suicidal attempt was assessed using the question: "During the past 12 months, how many times did you actually attempt suicide?". Response options included: "None", "1 time", "2-3 times", "4-5 times", "6 or more times", and suicide attempt was defined as

For peer review only - http://bmjopen.bmj.com/site/about/guidelines.xhtml

at least once in the past 12 months. Fighting was assessed by the question: “During the past 12 months, how many times were you in a physical fight?”. Answer options included “None”, “1 time”, “2-3 times”, “4-5 times”, “6-7 times”, “8-9 times”, “10-11 times”, “12 or more times”, and fighting was defined as at least once in the past 12 months. Being bullied was assessed by the question: “During the past 12 months, how many times has someone threatened or injured you with a weapon such as a gun, knife, or club on school property?”. Answer options included “None”, “1 time”, “2-3 times”, “4-5 times”, “6-7 times”, “8-9 times”, “10-11 times”, “12 or more times”. Being bullied was defined as being threatened or injured by someone at least once in the past 12 months. More detailed information on covariates is provided in **Table 1**.

Quality Control

The survey was conducted by trained surveyors from the local Center for Disease Control and Prevention. In order to improve response rate, every recruited student was given a pencil box as a gift, and the survey was anonymous.

Ethics Statement

The study design and procedure were approved by the ethics committee of Zhejiang Provincial Centre for Disease Control and Prevention. Written informed consent was obtained from all participants and their guardians before the survey.

Statistical analysis

All analyses were performed using SAS software V.9.3. A weighting factor was applied to each student record to adjust for non-response and for the varying probabilities of selection. The weight used for estimation in this survey is given by: W= W1×W2×f1×f2. W1 = the inverse of the probability of selecting the county. W2 = the inverse of the probability of selecting the classroom within the county. f1 = a student-level nonresponse adjustment factor calculated by class. f2 = a post-stratification adjustment factor calculated by grade[29]. Continuous variables were given as the mean ± standard deviation. The prevalence of current drinking and binge drinking was given as percent and 95% confidence intervals (CI). Weighted prevalence was calculated using the PROC SURVEYFREQ procedure and its difference between groups was compared using Rao-Scott Chi-square test. To assess the associations between each correlating factor and binge drinking, univariate and multivariable logistic regression analyses were performed using the PROC SURVEYLOGISTC procedure, to take into account the complex survey sampling methods. We first determined which factors were associated with binge drinking in univariate analyses (P<0.05), and variables significant in the univariate analyses were entered in a multivariable logistic regression model. All statistical tests were two tailed, and P-values <0.05 were considered statistically significant.

For peer review only

For peer review only - http://bmjopen.bmj.com/site/about/guidelines.xhtml

Results

Descriptive statistics

A total of 24 157 students were invited to participate. Due to missing or incomplete questionnaires and refusal to participate, 23 543 eligible participants (response rate 97.5%) were included in the current analyses. 12 068 (51.3%) participants were boys and, overall, mean age was 15.6 years. 12 207 (51.9%) participants were middle school students, 6 477 (27.5%) were academic high school students and 4 859 (20.6%) came from vocational high schools.

9.9% of students came from non-intact families (**Table 2**). 11.4% of students' paternal educational level was college or above. 17.7% of students' maternal educational level was middle or high school. 54.8% of students had siblings. 22.5% of students reported having excellent academic performance. 20.7% of students reported being physically active 6-7 days per week. 21.5% of students reported more than 4 hours screen-time per day. 64.1% of students reported never or occasionally feeling lonely during the past 12 months. 4.7% of students reported being often or always worried about something such that they could not sleep during the past 12 months. 5.5% of students reported smoking cigarettes during the past 30 days. 15.6% of students reported engaging in a physical fight, and 13.2% reported being bullied during the past 12 months. 3.9% of students reported ever having sexual intercourse.

The prevalence of current drinking and binge drinking

As shown in **Table 3**, the prevalence of current drinking was 22.8% (95%CI: 21.6-23.9), higher among boys than girls (27.0% vs. 18.2%), among students who ever attempted suicide (48.4% vs. 21.8% in those without suicide attempt), among current smokers (73.3% vs. 19.8% in non-smokers), and among students who reported ever engaging in a physical fight (40.3% vs. 19.5% in those without physical fight), ever being bullied (31.1% vs. 21.5% in those without being bullied), or having sexual experience (59.1% vs. 21.3% in those without sexual experience). There was no statistically significant difference between urban and rural areas (24.4% vs. 22.0%). In addition, the prevalence of current drinking increased with poorer academic performance, longer duration of screen-time, and higher severity of loneliness or insomnia (all P for trend <0.0001).

The prevalence of binge drinking was 9.2% (95%CI: 8.5-10.0) overall, and was 6.3%, 7.7% and 17.9%, respectively in middle school, academic high school, and vocational high school. The associations of binge drinking with sociodemographic and behavioural factors were similar to those of current drinking. The prevalence of binge drinking was higher among boys than among girls (11.8% vs. 6.5%), among students with previous suicide attempts (31.5% vs. 8.4%), among students who reported smoking cigarettes (51.2% vs. 6.8%), involvement in a physical fight (21.5% vs. 7.0%), being bullied (14.1% vs. 8.5%), previous sexual experience (39.2% vs. 8.1%), poor

For peer review only - http://bmjopen.bmj.com/site/about/guidelines.xhtml

academic performance, longer duration of screen-time, and higher severity of loneliness or insomnia (*P* for trend <0.0001 for the latter 4 parameters). There was no urban-rural difference in the prevalence of binge drinking (9.8% vs. 9.0%).

Logistic regression analysis

After adjusting for other variables included in the model, multivariable analysis showed that, compared to boys aged ≤13 years, older boys were more likely to binge drink (**Table 4**). Compared to boys in middle school, boys attending academic high school and vocational high school had 1.5 (OR=1.48, 95%CI: 1.15-1.90) and 2.1 (OR=2.09, 95%CI: 1.59-2.74) times higher probability of binge drinking. Girls attending vocational high school were 2.2 times more likely to binge drink in comparison to girls attending middle school (OR=2.18, 95%CI: 1.49-3.19). Boys with poor academic performance were 1.3 times more likely to binge drink in comparison to boys with excellent academic performance (OR=1.27, 95%CI: 1.03-1.56). Compared to boys who were not physically active within the past 7 days, boys who were physically active on 6-7 days had a higher risk of binge drinking (OR=1.33, 95%CI: 1.07-1.66). Compared to girls with screen-time of 0, girls with screen-time of 1-4 hours and more than 4 hours per day were 1.6 times (OR=1.61, 95%CI: 1.20-2.17) and 2.6 (OR=2.59, 95%CI: 1.93-3.48) times, respectively, more likely to binge drink. Boys with screen-time greater than 4 hours per day had a higher risk of binge drinking in comparison to boys with screen-time of 0 (OR=1.44, 95%CI: 1.16-1.79). Girls who often or always felt lonely were 1.4 times more likely to binge drink than girls who never or occasionally felt lonely (OR=1.43, 95%CI: 1.05-1.95). Boys who ever attempted suicide had 2.4 times higher odds of binge drinking than boys without a history of attempted suicide (OR=2.37, 95%CI: 1.73-3.25). The corresponding odds ratio for girls was 3.4 (OR=3.36, 95%CI: 2.48-4.56). Both boys and girls who reported smoking cigarettes within the past 30 days were 5.2 times (OR=5.21, 95%CI: 4.13-6.58) and 6.7 (OR=6.68, 95%CI: 4.64-9.60) times, respectively, more likely to binge drink than their counterparts who did not smoke cigarettes. Boys who reported being involved in physical fight in previous 12 months had a 2.2 times higher risk of binge drinking than those who reported not being involved in a physical fight (OR=2.18, 95%CI: 1.81-2.62). The corresponding figure for girls was 2.5 (OR=2.48, 95%CI: 1.87-3.29). Boys who had been bullied within the previous 12 months had a 1.2 times higher risk of binge drinking than boys who had not been bullied (OR=1.23, 95%CI: 1.03-1.46). Both boys and girls who had sexual experience were 2.3 times (OR=2.33, 95%CI: 1.69-3.20) and 1.8 (OR=1.82, 95%CI: 1.22-2.72) times, respectively, more likely to binge drink than their counterparts without sexual experience.

Discussion

In this study of middle and high school students in Zhejiang, China, we examined the prevalence of current drinking and binge drinking, and identified and quantified the associations of socio-

For peer review only - http://bmjopen.bmj.com/site/about/guidelines.xhtml

demographic and behavioural correlates of binge drinking, providing information to enable development of interventions to prevent binge drinking among this population group.

Prevalence of binge drinking

Due to different definitions of binge drinking, direct comparisons between studies are difficult. Since using a 5-drink measure for high school students in 1975, most national surveys have defined binge drinking as 5 or more drinks among both women and men[12 17]. A new gender-specific measure of ≥4/5 drinks for women/men has been used by Harvard School to reflect gender differences in the risk of alcohol-related harms[30-32]. The use of these gender-specific thresholds is also justified by women's generally smaller stature, and physiological differences between men and women affecting the absorption and distribution of alcohol[33]. Other studies have also used a 6-drink measure for both women and men[18]. In the present study, we adopted the definition of ≥4/5 drinks for women/wen and found an overall prevalence of binge drink in Zhejiang of 9.2%, which was higher than previously reported in Hong Kong (7.1%)[15]. The higher prevalence among boys than girls was consistent with results from other studies[15 18 34], but differs from a Korean study, in which no sex difference was observed[14]. The highest prevalence of binge drinking in the current study was among students attending vocational high schools. A possible explanation for this was that, compared to middle school and academic high school students, vocational high school students are likely to enter employment immediately following graduation, for which social communication and interaction, possibly associated with alcohol consumption, may be considered important.[17]. In subgroup analyses, the highest prevalence of binge drinking was among current drinkers (51.2%), students with sexual experience (39.2%), and students who had a history of a previous suicide attempt (31.5%). This suggests that such students should be identified as target populations for interventions to prevent and address binge drinking.

Association of demographic factors of binge drinking

No association was found between parental educational levels and binge drinking in our study. In a systematic review, including 20 studies from 10 countries or areas, parental socioeconomic status (SES), defined as the educational level, income, or occupation, was weakly positively associated with binge drinking in developing countries. However, no such association was not found in developed countries[35]. A previous study reported that non-intact family structure was associated with alcohol drinking among adolescents due to low family attachment or insufficient parent-child communication[24 25]. However, no such association was found in our study, which may reflect somewhat cultural differences in parental attitudes to alcohol consumption. Van den et al. found that adolescents were less likely to drink under strict alcohol-specific rules at homes despite non-intact families[36].

For peer review only - http://bmjopen.bmj.com/site/about/guidelines.xhtml

Association of behavioral factors of binge drinking

A cohort study, including 89 university students who were followed up for two years, found persistent binge drinking was associated with verbal memory and monitoring diffculties[37]. This might be a possible reason for the positive association we observed between poor academic performance and binge drinking (i.e. binge drinking may cause poor academic performance). Several earlier studies have also documented poorer performance among binge drinking students on neuropsychological tasks assessing inhibitory control, cognitive interference, sustained attention, verbal working memory and episodic declarative memory[38-41], functions which are known to be supported by prefrontal and/or hippocampal regions, and these may also explain associations between poor academic performance and binge drinking.

Buscemi et al. found a positive relationship between moderate physical activity and alcohol use among males, but not females[42]. Another study documented a positive relationship between vigorous physical activity and alcohol use, which was stronger at younger ages[43]. Our study showed that boys who were physically active were 1.3 times more likely to binge drink than boys who were not physically active. It is unclear whether physical activity leads to increased odds of binge drink or whether the converse is true (i.e. binge drinking causes increased physical activity). One possible hypothesis might be that more physically active boys may binge drink as a means of relaxation. Further prospective studies are warranted to ascertain the likely direction of association between physical activity and binge drinking, and underlying mechanisms.

In our study, nearly one in five students reported more than 4 hours screen-time per day. Previous studies have mainly focused on television watching as a potential risk factor for various diseases[44-47]. However, the exponential growth of electronic screen products suggests that focusing only on television viewing might underestimate screen-time[48]. To the best of our knowledge, this is the first study to examine the association between screen-time and adolescent binge drinking, finding a positive association between duration of exposure to electronic screen products and odds of binge drinking.

Alcohol drinking can damage neurons, decrease neurogenesis, and cause cognitive and affective dysfunction, especially among adolescents[49]. Laboratory evidence has shown that decreased neurogenesis results in depression-like behaviours in rats[50 51]. The relationship of alcohol use with mental health has been suggested to be bi-directional; Rohde et al. reported that the onset of psychiatric disorder preceded the onset of alcoholism[52], while Berglund et al. reported that depression followed alcoholism[53]. However, although a previous study has indicated that substance use might be a means of self-medication and of alleviating the negative feelings that emanate from being lonely,[54] binge drinking has been shown to be ineffective in improving mental health[55],. In our study, loneliness was positively associated with adolescent binge drinking, but this association was found only among girls, consistent with findings among

For peer review only - http://bmjopen.bmj.com/site/about/guidelines.xhtml

Arkhangelsk adolescents[56].Furthermore, Huang et al. reported that alcohol drinking was significantly associated with emotional symptoms among girls only[57]. Our findings suggest that preventive strategies against binge drinking for female adolescents should include mental health consultation.

As demonstrated in our study, with higher levels of insomnia, the odds of adolescent binge drinking was higher. A previous study by Popovici et al., including 14 089 participants, found that binge drinking was positively associated with sleep problems (having trouble falling asleep or staying asleep), independent of psychiatric conditions. There was a dose-response relationship between sleep problems and frequency of binge drinking[58], consistent with our study. In addition, Rishi et al. found that binge drinking could reverse sleep-wake cycle in rats and produce symptoms of insomnia [59], which could provide some explanation for the association between insomnia and binge drinking among students in the current study.

Alcohol drinking is a well-established risk factor for suicide attempts[60 61], and acute intoxication may be a stronger risk factor than chronic alcohol use[60], suggesting that binge drinking, which may produces rapid intoxication, may elevate the risk of suicide. In our study, prior suicide attempt was positively associated with binge drinking. Consistent with previous studies, binge drinking was related to the use of cigarettes[62 63]. In our study, current smoking had the strongest association with the odds of binge drinking, with over 5 and 6 times higher odds of binge drinking among current smoking boys and girls, respectively. Adolescents who drink alcohol are more likely to be involved in interpersonal conflicts and violence[23]. Our findings that both fighting and being bullied were positively related with adolescent binge drinking highlights the need for adolescent violence prevention programs focusing on the reduction of alcohol abuse. We found a positive association between binge drinking and sexual experience, which was consistent with an earlier study[23]. Lewis et al. reported that alcohol may facilitate formation of intimate relationship[64]. In addition, a previous study reported that binge drinking among adolescents was associated with higher rates of unwanted pregnancy, sexually transmitted infections, and infertility[8].

Limitations

Our study had several limitations. First, the a cross-sectional study design prevents establishment of the causal relationships between sociodemographic and behavioural factors and binge drinking. Second, all data was self-reported by students, and self-reported alcohol consumption may be susceptible to recall and social desirability biases. Third, only students attending schools participated in the survey. Students who had been expelled or suspended from school, or who stopped attending, may be more likely to binge drink, and the overall prevalence of binge drinking in our current study might therefore represent an underestimate of the true prevalence

For peer review only - http://bmjopen.bmj.com/site/about/guidelines.xhtml

For peer review only

Conclusions

Despite these limitations, our study identified correlates of binge drinking among middle and high school students in Zhejiang and quantified the strength of these associations, providing insight to inform binge drinking prevention strategies. Efforts to prevent binge drinking may need to address a cluster of correlating factors, including cigarette smoking, excessive screen-time, suicide attempt, fighting, being bullied, loneliness, insomnia and sexual behaviour. The presented findings provide evidence to assist healthcare providers in identifying students at high-risk of binge drinking, which will aid in the planning of prevention and intervention measures for at-risk students.

Acknowledgements The authors would like to thank all the students, parents, teachers and local officials for their participation, assistance and co-operation.

Contributor HW designed the study, and collected and analyzed the data with MY. JZ and RH were involved in data interpretation. HD and BF took part in data analysis and revised the manuscript. MW was involved in data collection. All the authors have read and approved the final submitted version.

Competing interests None declared.

Funding The work was supported by grant (2016YFC0900502) from National Key Research and Development Program of China.

Ethics approval Ethics Committee of Zhejiang Provincial Centre for Disease Control and Prevention.

Data sharing statement No additional data are available

References

1. Rehm J, Mathers C, Popova S, et al. Global burden of disease and injury and economic cost attributable to alcohol use and alcohol-use disorders. *Lancet* 2009;373(9682):2223-33. doi: 10.1016/S0140-6736(09)60746-7
2. Collaborators GBDRF. Global, regional, and national comparative risk assessment of 84 behavioural, environmental and occupational, and metabolic risks or clusters of risks, 1990-2016: a systematic analysis for the Global Burden of Disease Study 2016. *Lancet* 2017;390(10100):1345-422. doi: 10.1016/S0140-6736(17)32366-8
3. Bingham CR, Shope JT, Parow JE, et al. Crash types: markers of increased risk of alcohol-involved crashes among teen drivers. *J Stud Alcohol Drugs* 2009;70(4):528-35.
4. Scott KD, Schafer J, Greenfield TK. The role of alcohol in physical assault perpetration and victimization. *J Stud Alcohol* 1999;60(4):528-36.
5. Foran HM, O'Leary KD. Alcohol and intimate partner violence: a meta-analytic review. *Clin Psychol Rev* 2008;28(7):1222-34. doi: 10.1016/j.cpr.2008.05.001

For peer review only - http://bmjopen.bmj.com/site/about/guidelines.xhtml

6. Katon W, Richardson L, Russo J, et al. Depressive symptoms in adolescence: the association with multiple health risk behaviors. *Gen Hosp Psychiatry* 2010;32(3):233-9. doi: 10.1016/j.genhosppsych.2010.01.008
7. Marmorstein NR. Longitudinal associations between alcohol problems and depressive symptoms: early adolescence through early adulthood. *Alcohol Clin Exp Res* 2009;33(1):49-59. doi: 10.1111/j.1530-0277.2008.00810.x
8. Stolle M, Sack PM, Thomasius R. Binge drinking in childhood and adolescence: epidemiology, consequences, and interventions. *Dtsch Arztebl Int* 2009;106(19):323-8. doi: 10.3238/arztebl.2009.0323
9. Degenhardt L, O'Loughlin C, Swift W, et al. The persistence of adolescent binge drinking into adulthood: findings from a 15-year prospective cohort study. *BMJ Open* 2013;3(8):e003015. doi: 10.1136/bmjopen-2013-003015
10. Viner RM, Taylor B. Adult outcomes of binge drinking in adolescence: findings from a UK national birth cohort. *J Epidemiol Community Health* 2007;61(10):902-7. doi: 10.1136/jech.2005.038117
11. Kim JH, Lee S, Chow J, et al. Prevalence and the factors associated with binge drinking, alcohol abuse, and alcohol dependence: a population-based study of Chinese adults in Hong Kong. *Alcohol Alcohol* 2008;43(3):360-70. doi: 10.1093/alcalc/agm181
12. The ESPAD Group: ESPAD Report 2015 Result from the European School Survey Project on Alcohol and Other Drugs. Lisbon, 2016.
13. Esser MB, Clayton H, Demissie Z, et al. Current and Binge Drinking Among High School Students - United States, 1991-2015. *MMWR Morb Mortal Wkly Rep* 2017;66(18):474-78. doi: 10.15585/mmwr.mm6618a4
14. Chung SS, Joung KH. Risk factors of heavy episodic drinking among Korean adolescents. *J Psychiatr Ment Health Nurs* 2013;20(8):665-71. doi: 10.1111/j.1365-2850.2012.01954.x
15. Huang R, Ho SY, Wang MP, et al. Sociodemographic risk factors of alcohol drinking in Hong Kong adolescents. *J Epidemiol Community Health* 2016;70(4):374-9. doi: 10.1136/jech-2015-206418
16. Qian L, Yi HG, Tian BC, et al. [Alcohol consumption and drug use among middle school students aged 13-15 in 4 cities of China]. *Zhonghua Liu Xing Bing Xue Za Zhi* 2006;27(6):483-7.
17. Xing Y, Ji C, Zhang L. Relationship of binge drinking and other health-compromising behaviors among urban adolescents in China. *J Adolesc Health* 2006;39(4):495-500. doi: 10.1016/j.jadohealth.2006.03.014
18. Lu S, Du S, Hu X, et al. Drinking patterns and the association between socio-demographic factors and adolescents' alcohol use in three metropolises in China. *Int J Environ Res Public Health* 2015;12(2):2037-53. doi: 10.3390/ijerph120202037
19. Au WM, Ho SY, Wang MP, et al. Correlates of pro-drinking practices in drinking parents of adolescents in Hong Kong. *PLoS One* 2015;10(3):e0119554. doi: 10.1371/journal.pone.0119554
20. Wang S, Newman IM, Shell DF. Cultural Orientation and Its Associations with Alcohol Use by University Students in China. *PLoS One* 2016;11(11):e0165858. doi: 10.1371/journal.pone.0165858
21. Newman J, Xue J, Fang X. Alcohol Use and Its Risk Factors Among High School Students in Beijing *Chin J Sch Health* 2004;25(4):385-86.
22. Zuo J, Wang H, Zhou N. Investigation on the the Drinking State and the Influencing Factors in Middle School Students in Wuhan City. *Chin Med Soc* 2001;14(3):10-12.
23. Miller JW, Naimi TS, Brewer RD, et al. Binge drinking and associated health risk behaviors among high school students. *Pediatrics* 2007;119(1):76-85. doi: 10.1542/peds.2006-1517
24. Sokol-Katz J, Dunham R, Zimmerman R. Family structure versus parental attachment in controlling adolescent deviant behavior: a social control model. *Adolescence* 1997;32(125):199-215.

For peer review only - http://bmjopen.bmj.com/site/about/guidelines.xhtml

25. Mak KK, Ho SY, Thomas GN, et al. Family structure, parent-child conversation time and substance use among Chinese adolescents. *BMC Public Health* 2010;10:503. doi: 10.1186/1471-2458-10-503
26. Centers for Disease Control and Prevention . 2017 State and Local Youth Risk Behavior Survey; 2017 [Available from: https://www.cdc.gov/healthyyouth/data/yrbs/questionnaires.htm accessed 21/11 2017.
27. World Health Organization.Global school-based students health survey(GSHS) 2015 [updated 7/6. Available from: http://www.who.int/ncds/surveillance/gshs/nepal/en/ accessed 21/11 2017.
28. Newsletter N. NIAAA Council Approves Definition of Binge Drinking. 2004.
29. Pengpid S, Peltzer K. Alcohol use and associated factors among adolescent students in Thailand. *West Indian Med J* 2012;61(9):890-6.
30. Wechsler H, Austin SB. Binge drinking: the five/four measure. *J Stud Alcohol* 1998;59(1):122-4.
31. Wechsler H, Dowdall GW, Davenport A, et al. A gender-specific measure of binge drinking among college students. *Am J Public Health* 1995;85(7):982-5.
32. Wechsler H, Nelson TF. Binge drinking and the American college student: what's five drinks? *Psychol Addict Behav* 2001;15(4):287-91.
33. Baraona E, Abittan CS, Dohmen K, et al. Gender differences in pharmacokinetics of alcohol. *Alcohol Clin Exp Res* 2001;25(4):502-7.
34. Tavolacci MP, Boerg E, Richard L, et al. Prevalence of binge drinking and associated behaviours among 3286 college students in France. *BMC Public Health* 2016;16:178. doi: 10.1186/s12889-016-2863-x
35. Kwok KH, Yuan SN. Parental socioeconomic status and binge drinking in adolescents: A systematic review. *Am J Addict* 2016 doi: 10.1111/ajad.12461
36. van den Eijnden R, Mheen D, Vet R, et al. Alcohol-specific parenting and adolescents' alcohol-related problems: the interacting role of alcohol availability at home and parental rules. *J Stud Alcohol Drugs* 2011;72(3):408-17.
37. Mota N, Parada M, Crego A, et al. Binge drinking trajectory and neuropsychological functioning among university students: a longitudinal study. *Drug Alcohol Depend* 2013;133(1):108-14. doi: 10.1016/j.drugalcdep.2013.05.024
38. Heffernan T, Clark R, Bartholomew J, et al. Does binge drinking in teenagers affect their everyday prospective memory? *Drug Alcohol Depend* 2010;109(1-3):73-8. doi: 10.1016/j.drugalcdep.2009.12.013
39. Ehlers CL, Phillips E, Finnerman G, et al. P3 components and adolescent binge drinking in Southwest California Indians. *Neurotoxicol Teratol* 2007;29(1):153-63. doi: 10.1016/j.ntt.2006.11.013
40. Garcia-Moreno LM, Exposito J, Sanhueza C, et al. [Prefrontal activity and weekend alcoholism in the young]. *Adicciones* 2008;20(3):271-9.
41. Johnson CA, Xiao L, Palmer P, et al. Affective decision-making deficits, linked to a dysfunctional ventromedial prefrontal cortex, revealed in 10th grade Chinese adolescent binge drinkers. *Neuropsychologia* 2008;46(2):714-26. doi: 10.1016/j.neuropsychologia.2007.09.012
42. Buscemi J, Martens MP, Murphy JG, et al. Moderators of the relationship between physical activity and alcohol consumption in college students. *J Am Coll Health* 2011;59(6):503-9. doi: 10.1080/07448481.2010.518326
43. Lisha NE, Martens M, Leventhal AM. Age and gender as moderators of the relationship between physical activity and alcohol use. *Addict Behav* 2011;36(9):933-6. doi: 10.1016/j.addbeh.2011.04.003
44. Alghadir AH, Gabr SA, Iqbal ZA. Television watching, diet and body mass index of school children in Saudi Arabia. *Pediatr Int* 2016;58(4):290-4. doi: 10.1111/ped.12834
45. Al-Ghamdi SH. The association between watching television and obesity in children of school-age in Saudi Arabia. *J Family Community Med* 2013;20(2):83-9. doi: 10.4103/2230-8229.114767

For peer review only - http://bmjopen.bmj.com/site/about/guidelines.xhtml

46. Mamun AA, O'Callaghan MJ, Williams G, et al. Television watching from adolescence to adulthood and its association with BMI, waist circumference, waist-to-hip ratio and obesity: a longitudinal study. *Public Health Nutr* 2013;16(1):54-64. doi: 10.1017/S1368980012002832
47. Mekary RA, Lucas M, Pan A, et al. Isotemporal substitution analysis for physical activity, television watching, and risk of depression. *Am J Epidemiol* 2013;178(3):474-83. doi: 10.1093/aje/kws590
48. Jiang XX, Hardy LL, Ding D, et al. Recreational screen-time among Chinese adolescents: a cross-sectional study. *J Epidemiol* 2014;24(5):397-403.
49. Guerri C, Pascual M. Mechanisms involved in the neurotoxic, cognitive, and neurobehavioral effects of alcohol consumption during adolescence. *Alcohol* 2010;44(1):15-26. doi: 10.1016/j.alcohol.2009.10.003
50. Santarelli L, Saxe M, Gross C, et al. Requirement of hippocampal neurogenesis for the behavioral effects of antidepressants. *Science* 2003;301(5634):805-9. doi: 10.1126/science.1083328
51. Malberg JE, Eisch AJ, Nestler EJ, et al. Chronic antidepressant treatment increases neurogenesis in adult rat hippocampus. *J Neurosci* 2000;20(24):9104-10.
52. Rohde P, Lewinsohn PM, Seeley JR. Psychiatric comorbidity with problematic alcohol use in high school students. *J Am Acad Child Adolesc Psychiatry* 1996;35(1):101-9. doi: 10.1097/00004583-199601000-00018
53. Berglund M, Ojehagen A. The influence of alcohol drinking and alcohol use disorders on psychiatric disorders and suicidal behavior. *Alcohol Clin Exp Res* 1998;22(7 Suppl):333S-45S.
54. Page RM, Dennis M, Lindsay GB, et al. Psychosocial distress and substance use among adolescents in four countries: Philippines, China, Chile, and Namibia. *Youth & Society* 2011;43(3):900-30.
55. Kim JH, Chan KW, Chow JK, et al. University binge drinking patterns and changes in patterns of alcohol consumption among chinese undergraduates in a Hong Kong university. *J Am Coll Health* 2009;58(3):255-65. doi: 10.1080/07448480903295318
56. Stickley A, Koyanagi A, Koposov R, et al. Loneliness and health risk behaviours among Russian and U.S. adolescents: a cross-sectional study. *BMC Public Health* 2014;14:366. doi: 10.1186/1471-2458-14-366
57. Huang R, Ho SY, Wang MP, et al. Reported alcohol drinking and mental health problems in Hong Kong Chinese adolescents. *Drug Alcohol Depend* 2016;164:47-54. doi: 10.1016/j.drugalcdep.2016.04.028
58. Popovici I, French MT. Binge drinking and sleep problems among young adults. *Drug Alcohol Depend* 2013;132(1-2):207-15. doi: 10.1016/j.drugalcdep.2013.02.001
59. Sharma R, Bradshaw K, Sahota P, et al. Acute binge alcohol administration reverses sleep-wake cycle in Sprague Dawley rats. *Alcohol Clin Exp Res* 2014;38(7):1941-6. doi: 10.1111/acer.12463
60. Cherpitel CJ, Borges GL, Wilcox HC. Acute alcohol use and suicidal behavior: a review of the literature. *Alcohol Clin Exp Res* 2004;28(5 Suppl):18S-28S.
61. Kaplan MS, Giesbrecht N, Caetano R, et al. Acute alcohol consumption as a contributing factor to suicidal behavior. *Am J Public Health* 2013;103(9):e2-3. doi: 10.2105/AJPH.2013.301422
62. Hale D, Viner R. Trends in the prevalence of multiple substance use in adolescents in England, 1998-2009. *J Public Health (Oxf)* 2013;35(3):367-74. doi: 10.1093/pubmed/fdt022
63. Stickley A, Koyanagi A, Koposov R, et al. Binge drinking among adolescents in Russia: prevalence, risk and protective factors. *Addict Behav* 2013;38(4):1988-95. doi: 10.1016/j.addbeh.2012.12.009
64. Lewis MA, Granato H, Blayney JA, et al. Predictors of hooking up sexual behaviors and emotional reactions among U.S. college students. *Arch Sex Behav* 2012;41(5):1219-29. doi: 10.1007/s10508-011-9817-2

For peer review only - http://bmjopen.bmj.com/site/about/guidelines.xhtml

Table 1 questions comprising variables included in the survey

Variables	Questions and options
Parental education level	What is the highest level of education your father/mother has obtained? (Answer options: primary school or below, middle school, high school, college or university, master graduates or above, unknown)
Parental marital status	What is your parents' current marital status? (Answer options: married, divorced, widowed, separated)
Siblings	Are you the only son/daughter of your parent?(Answer options: yes, no)
Physical activity	During the past 7 days, on how many days were you physically active for a total of at least 60 minutes per day?(Answer options: none, 1 days, 2 days, 3 days, 4 days, 5 days, 6 days, 7 days)
Academic performance	How would you describe your grades in class?(Answer options: excellent, middle, poor)
Screen-time	On an average school day, how many hours do you play video or computer games or use a computer for something that is not school work? (Answer options: none, <1 h/d, 1 h/d, 2 h/d, 3 h/d, 4 h/d, ≥5 h/d)
Loneliness	During the past 12 months, did you ever feel lonely? (Answer options: never, occasional, sometimes, often, always)
Insomnia	During the past 12 months, have you ever felt worried about something such that you cannot fall asleep? (Answer options: never, occasional, sometimes, often, always)
Suicidal attempt	During the past 12 months, how many times did you actually attempt suicide? (Answer options: none, 1 time, 2-3times, 4-5 times, 6 or more times)
Current smoking	During the past 30 days, on how many days did you smoke cigarettes? (Answer options: none, 1-2 days, 3-5 days, 6-9 days, 10-19 days, 20-29 days and all 30 days)
Fighting	During the past 12 months, how many times were you in a physical fight？(Answer options: none, 1 time, 2-3times, 4-5 times, 6-7 times, 8-9 times, 10-11 times, 12 or more times)
Being bullied	During the past 12 months, how many times has someone threatened or injured you with a weapon such as a gun, knife, or club on school property? (Answer options: none, 1 time, 2-3times, 4-5 times, 6-7 times, 8-9 times, 10-11 times, 12 or more times)
Sexual experience	Have you ever had sexual intercourse? (Answer options: yes, no)

For peer review only - http://bmjopen.bmj.com/site/about/guidelines.xhtml

Table 2 characteristics of adolescents from Zhejiang (N=23543)

Characteristics	Total	Boys	Girls
Age range (years)			
≤13	5 159 (20.9)	2 689 (21.1)	2 470 (20.8)
14	4 300 (17.8)	2 192 (17.7)	2 108 (17.8)
15	3 730 (16.5)	1 905 (16.5)	1 825 (16.6)
≥16	10 354 (44.8)	5 282 (44.7)	5 072 (44.8)
Area			
Urban	9 022 (31.8)	4 544 (31.8)	4 478 (31.9)
Rural	14 521 (68.2)	7 524 (68.2)	6 997 (68.1)
Types of school			
Middle school	12 207 (51.8)	6 364 (52.4)	5 843 (51.0)
Academic high school	6 477 (26.1)	3 223 (25.1)	3 254 (27.3)
Vocational high school	4 859 (22.1)	2 481 (22.5)	2 378 (21.7)
Parental marital status			
Married	21 151 (90.1)	10 924 (90.9)	10 227 (89.2)
Others	2 392 (9.9)	1 144 (9.1)	1 248 (10.8)
Paternal education level			
Primary or below	13 568 (60.0)	6 908 (59.5)	6 660 (60.5)
Middle or high school	5 100 (20.8)	2 628 (20.8)	2 472 (20.7)
College or above	3 129 (11.4)	1 575 (11.2)	1 554 (11.7)
Unknown	1 746 (7.8)	957 (8.5)	789 (7.1)
Maternal education level			
Primary or below	14 530 (63.9)	7 292 (62.5)	7 238 (65.5)
Middle or high school	4 363 (17.7)	2 271 (18.0)	2 092 (17.3)
College or above	2 736 (10.0)	1 392 (9.8)	1 344 (10.2)
Unknown	1 914 (8.4)	1 113 (9.7)	801 (7.0)
Having siblings			
Yes	12 137 (54.8)	5 263 (46.6)	6 874 (63.8)
No	11 406 (45.2)	6 805 (53.4)	4 601 (36.2)
Academic performance			
Excellent	5 448 (22.5)	2 731 (21.7)	2 717 (23.3)
Middle	11 765 (50.5)	5 727 (48.1)	6 038 (53.1)
Bad	6 330 (27.0)	3 610 (30.2)	2 720 (23.6)
Physical activity (d/wk)			
0	4 883 (21.5)	2 079 (18.1)	2 804 (25.2)
1-2	5 690 (24.8)	2 703 (22.9)	2 987 (26.8)
3-5	8 050 (33.0)	4 237 (34.5)	3 813 (31.5)
6-7	4 920 (20.7)	3 049 (24.5)	1 871 (16.5)
Screen-time (h/d)			
0	7 255 (31.2)	3 604 (29.7)	3 651 (32.8)
<1	4 009 (15.9)	1 968 (15.1)	2 041 (16.7)
1-4	7 621 (31.4)	3 853 (31.1)	3 768 (31.6)
⩾4	4 658 (21.5)	2 643 (24.1)	2 015 (18.9)
Loneliness			
Never/occasional	15 122 (64.1)	8 082 (66.8)	7 040 (61.2)
Sometimes	5 783 (24.7)	2 698 (22.6)	3 085 (26.8)
Often/always	2 638 (11.2)	1 288 (10.6)	1 350 (12.0)
Insomnia			
Never/occasional	18 682 (78.6)	9 880 (81.2)	8 802 (75.8)
Sometimes	3 765 (16.7)	1 653 (14.3)	2 112 (19.3)
Often/always	1 096 (4.7)	535 (4.5)	561 (4.9)
Suicide attempt			
Yes	854 (3.6)	337 (2.9)	517 (4.4)
No	22 689 (96.4)	1 1731 (97.1)	10 958 (95.6)
Current smoking			
Yes	1 247 (5.5)	968 (8.2)	279 (2.5)
No	22 296 (94.5)	1 1100 (91.8)	11 196 (97.5)
Fighting			
Yes	3 707 (15.6)	2 966(24.1)	741 (6.4)
No	19 836 (84.4)	9 102(75.9)	10 734 (93.6)
Being bullied			
Yes	3 041 (13.2)	1 872(15.7)	1169 (10.5)
No	20 502 (86.8)	10 196(84.3)	10 306 (89.5)
Sexual experience			
Yes	861 (3.9)	591 (5.1)	270(2.5)
No	22 682 (96.1)	11 477 (94.9)	11 205(97.5)

Number in brackets were weighted proportion

For peer review only - http://bmjopen.bmj.com/site/about/guidelines.xhtml

Table 3 weighted prevalence of current drinking and binge drinking by different subgroups

Characteristics	Current drinking			Binge drinking		
	Prevalence (%)*	Chi-Square	*P*	Prevalence (%)*	Chi-Square	*P*
Sex		93.65†	<.0001		80.46†	<.0001
Boys	27.0 (25.6-28.5)			11.8 (10.8-12.9)		
Girls	18.2 (16.8-19.6)			6.5 (5.6-7.3)		
Area		2.38†	0.12		0.62†	0.4314
Urban	24.4 (21.9-26.9)			9.8 (8.2-11.4)		
Rural	22.0 (20.5-23.5)			9.0 (8.0-10.0)		
Types of school		319.93†	<.0001		278.29†	<.0001
Middle school	17.5 (16.2-18.8)			6.3 (5.6-7.0)		
Academic high school	22.1 (20.4-23.8)			7.7 (6.8-8.7)		
Vocational high school	35.9 (33.7-38.0)			17.9 (15.9-20.0)		
Academic performance		68.9‡	<.0001		51.7‡	<.0001
Excellent	18.2 (16.4-20.0)			6.9 (5.7-8.1)		
Middle	21.6 (20.2-22.9)			8.5 (7.6-9.3)		
Poor	28.8 (26.9-30.6)			12.7 (11.5-13.8)		
Physical activity (d/wk)		1.10‡	0.2977		2.0‡	0.1555
0	23.2 (21.6-24.8)			9.7 (8.7-10.8)		
1-2	22.0 (20.5-23.5)			8.5 (7.4-9.5)		
3-5	21.7 (20.1-23.2)			8.3 (7.3-9.3)		
6-7	25.0 (23.0-26.9)			11.2 (9.9-12.5)		
Screen-time (h/d)		330.9‡	<.0001		259.6‡	<.0001
0	16.1 (14.8-17.3)			5.5 (4.8-6.3)		
<1	16.8 (15.3-18.3)			5.5 (4.7-6.4)		
1-4	23.3 (21.8-24.7)			8.9 (7.8-9.9)		
≥4	36.1 (34.0-38.1)			17.9 (16.3-19.6)		
Loneliness		149.6‡	<.0001		113.9‡	<.0001
Never/occasional	19.6 (18.4-20.7)			7.5 (6.8-8.2)		
Sometimes	26.2 (24.4-27.9)			10.4 (9.4-11.5)		
Often/always	33.5 (31.4-35.6)			16.5 (14.7-18.3)		
Insomnia		177.2‡	<.0001		205.3‡	<.0001
Never/occasional	20.4 (19.3-21.5)			7.7 (7.0-8.3)		
Sometimes	29.3 (27.3-31.2)			13.0 (11.6-14.5)		
Often/always	39.2 (35.8-42.5)			22.7 (19.9-25.5)		
Suicide attempt		204.61†	<.0001		288.99†	<.0001
Yes	48.4 (44.2-52.6)			31.5 (27.4-35.6)		
No	21.8 (20.6-23.0)			8.4 (7.7-9.2)		
Current smoking		7335.31†	<.0001		3720.33†	<.0001
Yes	73.3 (70.5-76.0)			51.2 (47.2-55.2)		
No	19.8 (18.9-20.8)			6.8 (6.3-7.4)		
Fighting		257.16†	<.0001		307.89†	<.0001
Yes	40.3 (37.2-43.4)			21.5 (19.1-23.9)		
No	19.5 (18.5-20.6)			7.0 (6.3-7.6)		
Being bullied		49.13†	<.0001		38.81†	<.0001
Yes	31.1 (28.1-34.1)			14.1 (12.0-16.2)		
No	21.5 (20.4-22.6)			8.5 (7.8-9.2)		
Sexual experience		526.80†	<.0001		500.40†	<.0001
Yes	59.1 (54.5-63.6)			39.2 (33.8-44.5)		
No	21.3 (20.2-22.4)			8.1 (7.4-8.7)		

*Based on the weighted data. †: Rao-Scott Chi-Square; ‡: Trend for Chi-Square

For peer review only - http://bmjopen.bmj.com/site/about/guidelines.xhtml

Table 4 crude and adjusted odd ratio of factors associated with binge drinking among adolescents in China

Variable	Boys (N=12 068)		Girls (N=11 475)	
	COR(95%CI)	AOR(95%CI)	COR(95%CI)	AOR(95%CI)
Age groups (ref: ≤13 years)				
14	**1.95 (1.52-2.50) &**	**1.95 (1.51-2.53) &**	1.12 (0.77-1.65)	1.16 (0.76-1.77)
15	**2.70 (2.06-3.53) &**	**2.21(1.67-2.90) &**	**1.46 (1.01-2.12) #**	1.12 (0.74-1.69)
≥16	**3.89 (3.07-4.92) &**	**2.10(1.55-2.84) &**	**1.68 (1.13-2.50) ***	1.03 (0.60-1.77)
Rural (ref: Urban)	1.00 (0.77-1.29)		0.77 (0.57-1.04)	
Types of school (ref: Middle school)				
Academic high school	**1.62 (1.33-1.96) &**	**1.48(1.15-1.90) ***	0.78 (0.60-1.00)	0.99 (0.64-1.55)
Vocational high school	**3.65 (2.91-4.58) &**	**2.09(1.59-2.74) &**	**2.71 (2.07-3.55) &**	**2.18 (1.49-3.19) $**
Parental marital status (ref: Married)				
Others	**1.32 (1.08-1.61) ***	0.91(0.73-1.13)	**2.07 (1.67-2.57) &**	1.25 (0.95-1.65)
Paternal education level				
(ref: Primary or below)				
Middle or high school	1.00 (0.84-1.20)	1.13(0.93-1.38)	0.84 (0.68-1.05)	1.01 (0.80-1.27)
College or above	**0.76 (0.60-0.95) #**	0.94(0.75-1.18)	**0.66 (0.49-0.90) ***	0.86 (0.62-1.19)
Unknown	0.91 (0.72-1.16)	0.99(0.78-1.26)	1.23 (0.89-1.71)	1.25 (0.88-1.79)
Maternal education level				
(ref: Primary or below)				
Middle or high school	0.99 (0.84-1.17)		0.99 (0.77-1.27)	
College or above	0.78 (0.61-1.00)		0.77 (0.56-1.05)	
Unknown	0.82 (0.64-1.06)		1.11 (0.77-1.61)	
Siblings (ref: No)	0.92 (0.80-1.06)		0.99 (0.80-1.22)	
Academic performance (ref: Excellent)				
Middle	**1.23 (1.01-1.50) #**	1.13 (0.93-1.36)	1.28 (0.95-1.73)	1.17 (0.87-1.58)
Bad	**1.82 (1.49-2.22) &**	**1.27 (1.03-1.56) #**	**1.97 (1.43-2.70)** &	1.30 (0.94-1.79)
Physical activity (ref: 0 d/wk)				
1-2 d/w	0.82 (0.66-1.02)	0.90 (0.70-1.16)	0.86 (0.70-1.06)	
3-5 d/w	**0.79 (0.65-0.96) #**	0.93 (0.75-1.14)	0.78 (0.60-1.02)	
6-7 d/w	1.09 (0.90-1.33)	**1.33 (1.07-1.66) #**	0.95 (0.74-1.21)	
Screen-time (ref: 0 h/d)				
<1 h/d	0.90 (0.72-1.12)	0.94 (0.75-1.17)	1.27 (0.88-1.84)	1.18 (0.81-1.73)
1-4 h/d	**1.47 (1.19-1.82) $**	1.14 (0.91-1.43)	**2.13 (1.62-2.80) &**	**1.61 (1.20-2.17) ***

For peer review only

≥ 4 h/d	**2.79 (2.28-3.41) &**	**1.44 (1.16-1.79) $**	**5.91 (4.53-7.71) &**	**2.59 (1.93-3.48) &**
Loneliness (ref: Never/Occasionally)				
Sometimes	**1.42 (1.23-1.63) &**	1.08 (0.91-1.28)	**1.69 (1.37-2.08) &**	1.23 (0.96-1.57)
Often/Always	**2.25 (1.87-2.72) &**	1.18 (0.90-1.56)	**3.14 (2.51-3.93) &**	**1.43 (1.05-1.95) #**
Insomnia (ref: Never/Occasionally)				
Sometimes	**1.97 (1.68-2.32) &**	**1.41 (1.13-1.77) ***	**1.92 (1.59-2.31) &**	**1.33 (1.06-1.65) $**
Often/Always	**2.89 (2.29-3.64) &**	**1.69 (1.23-2.31) ***	**5.18 (4.05-6.62) &**	**2.23 (1.63-3.06) &**
Suicide attempt (ref: No)	**4.14 (3.24-5.30) &**	**2.37 (1.73-3.25) &**	**7.35 (5.51-9.81) &**	**3.36 (2.48-4.56) &**
Current smoking (ref: No)	**11.24 (9.13-13.84) &**	**5.21 (4.13-6.58) &**	**20.27 (15.32-26.83) &**	**6.68 (4.64-9.60) &**
Fighting (ref: No)	**2.78 (2.37-3.27) &**	**2.18 (1.81-2.62) &**	**5.07 (4.04-6.37) &**	**2.48 (1.87-3.29) &**
Being bullied (ref: No)	**1.59 (1.34-1.89) &**	**1.23 (1.03-1.46) #**	**1.81 (1.27-2.59) ***	1.04 (0.69-1.56)
Sexual experience (ref: No)	**6.92 (5.33-8.98) &**	**2.33 (1.69-3.20) &**	**6.60 (4.94-8.83) &**	**1.82 (1.22-2.72) ***

Bold numbers represent significant results

COR: Crude odds ratio. AOR: Adjusted odds ratios. CI: confidence intervals. #: *P*<0.05. *: *P*<0.01. $: *P*<0.001. &: *P*<0.0001

AOR is adjusted for all other covariates in the model

For peer review only

For peer review only - http://bmjopen.bmj.com/site/about/guidelines.xhtml
